# Herbicides in Use: Current Status and Perspectives in the Different Biogeographic Regions of Europe

**DOI:** 10.1021/acs.jafc.5c03867

**Published:** 2025-08-26

**Authors:** Agnieszka Synowiec, Marta Czekaj, Mercedes Verdeguer, Diego G. De Barreda, Claudia Campillo-Cora, Yedra Vieites-Álvarez, David López-González, Adela M. Sánchez-Moreiras, David Fernández-Calviño, Fabio F. Nocito, Carla Ragonezi, Miguel A. Almeida Pinheiro de Carvalho, Merit Sutri, Merrit Shanskiy, Sigrún Dögg Eddudóttir, Tatiana P. Fedoniuk, Andrea Vityi, Ursula Bürgener, Liliana Piron, Mihai Gidea, Francisco Espinosa Escrig, Gülçin Beker Akbulut, Alicia Morugán Coronado, Esther Valiño, Fabrizio Araniti

**Affiliations:** † Department of Agroecology and Plant Cultivation, University of Agriculture in Kraków, Al. Mickiewicza 21, 31-120 Kraków, Poland; ‡ Department of Management and Economics of Enterprises, University of Agriculture in Kraków, Al. Mickiewicza 21, 31-120 Kraków, Poland; § Instituto Agroforestal Mediterráneo, 16774 Universitat Politècnica de València, Camino de Vera s/n, 46022 Valencia, Spain; ∥ Área de Edafoloxía e Química Agrícola, Departamento de Bioloxía Vexetal e Ciencia do Solo, Facultade de Ciencias, Universidade de Vigo, As Lagoas s/n, 32004 Ourense, Spain; ⊥ Instituto de Agroecoloxía e Alimentación (IAA), Universidade de Vigo, Campus Auga, 32004 Ourense, Spain; # Departamento de Bioloxía Vexetal e Ciencias do Solo, Facultade de Bioloxía, Universidade de Vigo, Campus Lagoas-Marcosende s/n, 36310 Vigo, Spain; ¶ Dipartimento di Scienze Agrarie e Ambientali—Produzione, Territorio, Agroenergia, 9304 Università degli Studi di Milano, 20133 Milano, Italy; ∇ ISOPlexis Centre Sustainable Agriculture and Food Technology, 56057 University of Madeira, Campus da Penteada, 9020-105 Funchal, Portugal; ○ Centre for the Research and Technology of Agro-Environmental and Biological Sciences (CITAB), Inov4Agro-Institute for Innovation, Capacity Building and Sustainability of Agri-Food Production, University of Trás-os-Montes and Alto Douro, 5000 Vila Real, Portugal; ⧫ Chair of Soil Science, Institute of Agricultural and Environmental Sciences, 85334 Estonian University of Life Sciences, Tartu 51006, Estonia; †† Icelandic Agricultural Advisory Center (RML), Höf∂́abakki 9, 110 Reykjavík, Iceland; ‡‡ Polissia National University, 10008 Zhytomyr, Ukraine; §§ 54610 University of Sopron, 9400 Sopron, Hungary; ∥∥ Agrarunternehmen Starbach-Sachsen eG (ASS), 01683 Nossen, Sachsen, Germany; ⊥⊥ League of Agricultural Producers Associations in Romania (LAPAR), 012244 Bucarest, Romania; ## SEIPASA S.A., Carrer Ciudad Dario, L’Alcúdia, 46250 Valencia, Spain; ¶¶ Department of Park and Ornamental Plants, 531771 Malatya Turgut Ozal University, 44210 Malatya, Turkiye; ∇∇ Sustainable Use, Management and Reclamation of Soil and Water Research Group, 16769 Universidad Politécnica de Cartagena, Paseo Alfonso XIII, 48, 30203 Cartagena, Spain; ○○ Fundación Empresa Universidad Gallega (FEUGA), 15705 Santiago de Compostela, Espana

**Keywords:** European herbicide regulations, chemical weed control, weed management, herbicide impacts, herbicide resistance, sustainable agriculture

## Abstract

This review examines the use of herbicides across Europe’s biogeographical regions, focusing on their historical development, regulatory framework, and environmental impacts. Since the 20th century, the use of herbicides has significantly increased agricultural productivity. However, the continuous use of herbicides with the same mode of action can lead to the development of resistant weeds, especially when low-diversity weed management strategies are employed. The European Union has established a strict approval process for herbicidal substances to safeguard environmental and human health. Consequently, the number of authorized active ingredients has declined due to concerns over their adverse effects. This review highlights the need for new sustainable tools for weed control and advocates reassessing Europe’s dependence on chemical herbicides, encouraging integrated weed management approaches and policies that balance productivity with environmental protection for a sustainable agricultural future.

## Graphical abstract

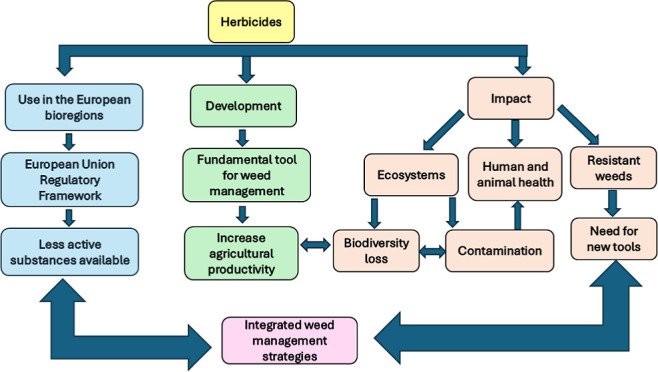

## 1 Introduction

Herbicides play a crucial role in modern weed management, significantly enhancing agricultural productivity and economic sustainability. Their use effectively reduces weed density and biomass, resulting in improved crop growth and higher yields.^1^ The introduction of selective herbicides in the late 1940s provided farmers with a new tool, enabling them to consider weed control more independently of their crop production system.^1,2^ The history of herbicide use and the main changes in weed management they caused highlight the importance of understanding the impact of herbicides on agricultural practices,^3^ both positive and negative. Without a doubt, the development of herbicides led to a significant increase in agricultural production worldwide and helped improve farmers’ incomes because it made weed management easier than ever before in the long history of agriculture.^4^

On the other hand, the intensive and improper use of herbicides has caused several environmental burdens over time. First, adverse direct impacts of herbicide use on weeds and habitat diversities and indirect effects on wildlife were noted.^5^ The increased use of agrochemicals and changes in agricultural land use, sped up by a decrease in crop diversities, simplified rotations, or even the predominance of crop monocultures, especially in central and northwestern Europe, have led to the selection of a larger group of aggressive weeds adapted to habitats with intermediate fertility.^6^ That also caused many less competitive weed species to become endangered or even extinct. Changes in weed communities caused a decline in food chains in the agroecosystems, especially in the composition of invertebrates, and a decrease in bacterial and fungal count in soils.^7^ These negative effects of herbicides were combined with other agronomic practices like mineral fertilization^8^ or soil tillage.^9^ Additionally, the broad use of herbicides has increased the risk of herbicide spray drift to surrounding vegetation,^10^ especially for some types of herbicide formulations (i.e., ester), nozzle selection, or application under wrong weather conditions.

Moreover, reliance on herbicides with the same mode of action, lacking the integration of alternative weed control methods in the agricultural system, has led to the selection of resistant weed populations.^11^ This issue has been a very urgent concern in agriculture for decades, despite scientists predicting the emergence of herbicide-resistant weeds shortly after their introduction.^12^ Also, the potential for life-history trade-offs associated with herbicide resistance emphasizes the need to understand the fitness of resistant weed populations.^13,14^ Some research shows that a fitness-cost penalty^15^ can sometimes offset the advantage of the resistance conferred by a mutation; however, more research is needed to measure the dominance of the resistance cost in the evolution of resistance.^16,17^

Since its commercial introduction in 1974, glyphosate has become the dominant herbicide worldwide^3^ and is also the most widely used herbicide in European countries,^18^ it can be applied in different types of annual and perennial^19^ crops and in nonagricultural areas like railway tracks and roadways.^20,21^ Additionally, it is used to terminate cover crops or temporary grassland. Recently, the new regulation 2023/2660 implemented in 2023 by the European Commission, renewing the approval for the use of glyphosate, has forbidden its use as preharvest crop desiccant to prevent the presence of residues on crop yield.^22^

In world agriculture, introducing Genetically Modified (GM) crops even speeded up glyphosate use by introducing glyphosate-resistant crops. Initially, it was seen as leading to the application of fewer and more benign herbicides.^19,23^ However, it finally resulted in massive and out-of-control use of this herbicide in those countries where glyphosate-resistant crops were allowed, such as Argentina, Brazil, USA, etc. In Europe, herbicide-tolerant GM crops are not allowed to be grown. However, Europe introduced mutant herbicide-tolerant crops, with the intensified use of the related herbicides—sulfonylureas, imidazolinones, and cyclohexanediones, which has sparked considerable debate, with conflicting claims about their ecological consequences.^24,25^

The use of herbicides has become a significant topic of debate and concern in the European Union, resulting in increasingly strict regulations (Directive 2009/128/EC, Commission Regulation (EU) No 284/2013). The European Union has limited or banned several herbicides due to their potential toxicity to aquatic ecosystems,^26^ prohibited or complicated herbicide application in certain European regions,^27^ and limited availability of alternative herbicides in the European Pesticides Database.^28^ Despite a European political consensus for significantly reducing herbicide use,^29^ concerns about this policy have shown competing interests between different actors involved in the agricultural use of pesticides.^30,31^ Farmers are compelled to reduce herbicide use to limit its impact on human health and the environment. However, most are concerned about the lack of effective alternatives^28,32^ or a decrease in revenue.^33^ At the same time, it is believed that a reduction of 50% in the use of pesticides would involve great changes in cultivation systems toward integrated production systems,^34^ which are more sustainable for the environment and can mitigate the financial risks associated with herbicide dependence.^28^

### 1.1 European Union Registration and Approval Process for Herbicides

The European Union (EU) has created a stringent and complex registration and approval process for herbicides, serving as a valuable model for global harmonization.^35^ The EU employs a Tier I risk assessment approach, achieving greater toxicity reduction for certain insecticides than herbicides and fungicides.^36^ As one of the strictest regulatory systems worldwide, the EU utilizes intricate yet flexible laws for pesticide regulation.^37^ The approval process includes rigorous user competency tests, maximum residue limits, and ongoing postregistration monitoring.^38^

Integrating epidemiological data into pesticide risk assessments is vital for the peer review of active substances seeking EU approval.^39^ The regulatory uncertainty in the EU is exemplified by the glyphosate controversy, which led to the extension of glyphosate’s authorization for another ten years despite significant opposition from the European Parliament. This situation highlights the need for independent research into the health effects of herbicides.^40,41^ The EU risk assessment process also requires studies on pesticide-active substances’ chronic and sublethal effects on honeybees, demonstrating a commitment to environmental protection and safety. Some studies reported glyphosate levels of 10 and 40 μg/kg in nectar and pollen collected by bees and over 100 other active substances, primarily insecticides.^42^ This raises concerns about the cumulative impact of these chemicals on bee populations and, subsequently, on biodiversity and food security.

The EU has established regulations for biopesticides that are as stringent as those for synthetic active substances, further enhancing consumer safety.^43^ Additionally, because the registration process can be complex, the EU offers guidance to help applicants and evaluators generate and assess the required data.^44^ The EU’s annual report also evaluates pesticide residue levels in foods available in the European market, highlighting its commitment to food safety.^45^

This regulatory framework involves a collaborative effort among three main stakeholders: the European Food Safety Authority (EFSA), the European Commission, and individual Member States. The process is organized into distinct steps, each critical to ensuring the safe use of pesticides within the EU. These steps include the initial application for authorization, scientific evaluation of data submitted by applicants, public consultations, and final decision-making, all aimed at maintaining high standards in agricultural safety.^46^

Companies seeking approval for active substances submit detailed applications to the relevant Member State. These applications contain extensive scientific data and studies, whether introducing new active substances or renewing or amending previously approved ones. Once the applications are submitted, the Member State conducts a thorough review and prepares an assessment report, which is forwarded to EFSA for further evaluation and risk assessment. The EFSA plays a crucial role in this process by providing independent scientific advice, ensuring that all potential risks to human health and the environment are meticulously evaluated before any pesticide can be authorized for use.^46^

After submission, the Member State thoroughly evaluates the application, assessing its scientific validity and potential risks. EFSA then performs a peer review of the Member State’s assessment in collaboration with other Member States. This review involves a detailed examination of the assessment report, ultimately resulting in scientific conclusions submitted to the European Commission. These conclusions may include recommendations for risk management measures and serve as a basis for deciding on the authorization of the active substance.^46^

Following EFSA’s review, the European Commission and Member States deliberate on granting authorization for the active substance. This decision-making process includes a proposal from the Commission for approval or rejection, followed by a vote from a special committee composed of Member State representatives. Typically, authorization for new active substances is granted ten years, while renewal applications may be extended to 15 years.^46^

Once an active substance is authorized, companies can apply for market placement of pesticides containing that substance. This application outlines the pesticide’s intended uses, including specific crops and application rates. The receiving Member State evaluates the application and proposes maximum residue levels (MRLs) as necessary.

If the proposed MRL complies with existing legislation, the application will be advanced to the European Commission for consideration. However, if the proposed MRL deviates from established norms, EFSA will conduct a comprehensive assessment and provide an opinion to the Commission. Ultimately, the Commission decides whether to accept the proposed MRL, determining the pesticide’s authorization status.^46^

Upon acceptance, the Member State can authorize the pesticide while adhering to EU regulations that govern pesticide authorization. Notably, the EU functions under three distinct zones for pesticide authorization, facilitating mutual recognition among Member States with similar agricultural practices.^46^

### 1.2 Environmental and Safety Considerations on Herbicides in Europe

Using herbicides in Europe has raised significant environmental and safety concerns, prompting regulatory measures and exploring alternative weed management strategies. Strict registration and environmental regulations have led to the loss of some herbicides in Europe, reflecting the region’s commitment to environmental protection.^12^ However, the loss of a single herbicide sometimes means losing the complete mode of action, as recently happened in Spain, when the HRAC/WSSA modes of action 22 and 23 were lost by only losing two herbicides, diquat, and chlorpropham, and it can increase the risk of herbicide-resistance evolution. Weedy plant species that have evolved resistance to herbicides due to enhanced metabolic capacity to detoxify herbicides (metabolic resistance), posing threats to herbicide sustainability and global crop production,^47^ are a major issue. Recent trends in herbicide regulation and registration include providing more detailed information to users to adjust rates according to prevailing environmental conditions and herbicide sensitivity with the primary regulatory objective of promoting the application of products at the recommended rate.^48^

The impact of herbicides on aquatic ecosystems has also been a concern, and a reliable herbicide hazard and risk assessment are necessary. An extensive catch-up must be made concerning macrophytes, the marine environment, and sediment as overlooked and understudied environmental compartments.^26^ Additionally, the ecological impact assessment of herbicides on aquatic floating vascular plants and freshwater species of phytoplankton has highlighted acute sensitivity and potential risks to these organisms.^49–52^

In response to these concerns in Europe, there is a growing interest in environmentally friendly weed management systems, where synthetic pesticides are reduced or even eliminated, resulting in considerable benefits in terms of biodiversity and soil conservation.^53,54^ The nonchemical methods include the use of different new mulch materials (paper, plastic, hydro-mulch, etc.),^55^ new thermal methods (laser diodes, microflames and capacity coupling of electric fields),^56^ sensor-based mechanical methods (image analysis with camera, global navigation satellite systems, laser and ultrasonic systems, all combined with mechanical systems),^57^ and the use of botanical herbicides (natural compounds such as carvacrol, thymol, eugenol, *p*-cymene, citral, etc.).^58^

European agriculture is highly diverse, shaped by variations in environmental conditions, climate, and socio-cultural factors. We hypothesize that these differences could influence herbicide consumption in agriculture. Therefore, this study aims to outline the current status of herbicides registered in the EU market and analyze herbicide consumption from 2016 to 2021 in selected European countries representing the 11 biogeographical regions of Europe (European Environment Agency, 2016).^59,60^ The biogeographical regions of Europe illustrate the continent’s diverse conditions, categorized into a hierarchical system based on their biotas.^60,61^ Europe is divided into 11 distinct biogeographical regions: Arctic, Boreal, Alpine, Continental, Atlantic, Pannonian, Steppic, Anatolian, Black Sea, Mediterranean, and Macaronesian. Understanding these regions is essential for conducting large-scale ecological analyses and developing effective conservation and management practices. In this context, it is interesting to examine the differences in herbicide use across these regions, as usage can vary based on a country’s geographic location, whether north to south or east to west. These differences are often correlated with the nature and role of agricultural production and environmental conditions.

## 2 Methods

The database of active substances, encompassing herbicides, insecticides, and fungicides authorized for use within the European Union, was extracted from the European Commission’s “Pesticide Database—Active Substance” Web site.^62^ This resource details each chemical compound, including approval and expiration dates and human and environmental toxicity profiles. It also indicates the EU member state/s where these substances are allowed. An initial screening aimed to distinguish herbicides from other pesticide categories within the vast inventory of 1485 chemicals. This led to the exclusion of substances no longer approved for use, narrowing down to a refined selection. The selected approved herbicidal substances were cataloged with specific information, such as molecular formulas, harmonized chemical classifications according to EUROSTAT (The complete data set detailing the herbicides is presented in Table S1), HRAC/WSSA modes of action (https://hacglobal.com),^63^ the state of herbicide resistance,^64^ and World Health Organization (WHO) hazard classes of herbicides.^65^

Herbicide consumption data (in tons of active ingredients per country) and the total area of cropland (in hectares) for the period 2016 to 2021 were collected for a set of countries representing various European biogeographical regions, including Slovakia (Alpine), Poland (Continental), Italy (Mediterranean), Hungary (Pannonian), Belgium (Atlantic), Türkiye (Anatolian & Black-Sea), Romania (Steppic), Estonia (Boreal), and Iceland (Arctic). This information was sourced from FAOSTAT^66^ and EUROSTAT.^67^ A brief description of the agricultural characteristics of each of the countries, based on statistical data, is presented in Table 1. Additionally, herbicide consumption data were compiled for different chemical families as outlined by EUROSTAT^67^ (Table 2—The complete data set detailing the herbicides is presented in Table S1), which enabled the calculation of each country’s herbicide used per hectare of cropland. According to the FAO, cropland is defined as land allocated for the cultivation of crops, including arable land and permanent crops. By comparing this data with the information on the European Commission’s “Pesticide Database–Active Substance” Web site,^62^ an analysis was conducted to ascertain the usage patterns of different chemical groups of herbicides in European bioregions.

**Table 1:** Characteristics of Agriculture in the Countries Representing the Biogeographical Regions

country/bioregion	utilized agricultural area (ha)^a^	arable land (ha)^a^	share of arable area in the utilized agricultural area (%)	orchards (ha)^b^	share of orchards in the utilized agricultural area (%)	average farm area (ha)^a^	main crops
Belgium/Atlantic	1,368,120	869,280	63.54	14,730	1.08	38.00	sugar beets, chicory, flax, cereal grains and potatoes
Estonia/Boreal	975,320	692,860	71.04	5141	0.52	85.78	winter wheat, spring barley, spring wheat, oilseed rape, oat
Hungary/Pannonian	4,921,740	4,027,970	81.84	36,292.48	0.74	21.21	wheat, maize, sunflower, rape, vineyards, fruit trees and berries
Iceland/Arctic^c^	91,531	12,902	0.14	0	0		forage crops, grass, barley, potatoes, turnips, carrots
Italy/Mediterranean	12,041,230	7,197,650	59.78	1,389,829.43	11.54	14.5	cereal grains, vine, olive, citrus fruits, fruit trees, vegetables, permanent meadows and pastures
Poland/Continental	14,749,240	11,147,160	75.58	167,314.99	1.13	11.33	winter wheat, oilseed rape, maize, apples
Romania/Steppic	12,762,830	8,570,730	67.15	67,840.29	0.53	4.42	winter wheat, oilseed rape, sunflower, maize, barley, soybean, apple, pear
Slovakia/Alpine	1,862,650	1,325,330	71.15	2321.44	0.12	94.89	wheat, barley, maize, oil crops, potatoes, sugar beet, vineyards, fruit trees
Turkey/Anatolian & Black Sea	38,089,000^d^	19,881,000^d^	52,20	3,694,255.7	15.43	6.1	wheat, sugar beet, cotton, vegetables and fruit

a EUROSTAT.^61^

b This category consist of Dessert apple trees, apple trees plantation for industrial processing, dessert pear trees, Pear trees for industrial processing, dessert peach and nectarine trees, peach and nectarine trees for industrial processing (including group of Pavie), apricot trees, orange trees, small citrus fruit trees, lemon trees, olive trees, table grape vines.

c RML (2024).^115^ Data for year 2020.

d FAOSTAT.^60^

**Table 2:** Table of the Herbicides Allowed in Europe According to the EU Pesticide Database^a^

herbicide	MoA	harmonised FAO classification	herbicide resistance cases [Heap 2024 ^58^	exp. approval
clethodim	HRAC/WSSA 1	cyclohexanedione herbicides	34 cases, 12 countries, 16 species	31/08/2026
clodinafop		aryloxyphenoxy-propionic herbicides	78 cases, 25 countries, 13 species	15/12/2025
cycloxydim		cyclohexanedione herbicides	26 cases, 10 countries, 9 species	31/08/2026
cyhalofop-butyl		aryloxyphenoxy-propionic herbicides	25 cases, 13 countries, 9 species	30/06/2032
diclofop		aryloxyphenoxy-propionic herbicides	87 cases, 20 countries, 13 species	31/08/2026
fenoxaprop-*P*-ethyl		aryloxyphenoxy-propionic herbicides	122 cases, 35 countries, 26 species	15/08/2025
fluazifop-*P*-butyl		aryloxyphenoxy-propionic herbicides	55 cases, 16 countries, 23 species	31/05/2026
pinoxaden		phenylpyrazole herbicides	60 cases, 18 countries, 16 species	30/06/2026
propaquizafop		aryloxyphenoxy-propionic herbicides	13 cases, 9 countries, 10 species	28/02/2027
quizalofop-*P*-ethyl		aryloxyphenoxy-propionic herbicides	40 cases, 15 countries, 20 species	28/02/2027
amidosulfuron	HRAC/WSSA 2	sulfonylurea herbicides	5 cases, 5 countries, 5 species	15/08/2025
bensulfuron		sulfonylurea herbicides	58 cases, 13 countries, 31 species	15/08/2026
flazasulfuron		sulfonylurea herbicides	5 cases, 3 countries, 4 species	31/07/2032
florasulam		anilide herbicides	36 cases, 17 countries, 23 species	31/12/2030
foramsulfuron		sulfonylurea herbicides	27 cases, 11 countries, 16 species	31/05/2035
halosulfuron-methyl		sulfonylurea herbicides	25 cases, 5 countries, 20 species	05/08/2025
imazamox		imidazolinone herbicides	74 cases, 18 countries, 43 species	31/01/2025
iodosulfuron-methyl		sulfonylurea herbicides	118 cases, 31 countries, 40 species	31/03/2032
mesosulfuron-methyl		sulfonylurea herbicides	89 cases, 24 countries, 26 species	30/06/2032
metsulfuron-methyl		sulfonylurea herbicides	84 cases, 18 countries, 40 species	31/03/2024
nicosulfuron		sulfonylurea herbicides	59 cases, 18 countries, 27 species	31/03/2027
penoxsulam		amide herbicides	29 cases, 13 countries, 14 species	15/05/2026
propoxycarbazone		triazolone herbicides	18 cases, 10 countries, 12 species	31/08/2032
prosulfuron		sulfonylurea herbicides	8 cases, 4 countries, 7 species	31/07/2024
pyroxsulam		amide herbicides	56 cases, 19 countries, 27 species	30/04/2025
rimsulfuron (aka renriduron)		sulfonylurea herbicides	17 cases, 9 countries, 12 species	15/08/2025
sulfosulfuron		sulfonylurea herbicides	22 cases, 12 countries, 14 species	31/12/2030
thiencarbazone-methyl		triazolone herbicides	6 cases, 4 countries, 5 species	30/09/2024
thifensulfuron-methyl		sulfonylurea herbicides	93 cases, 13 countries, 31 species	31/10/2031
tribenuron (aka metometuron)		sulfonylurea herbicides	105 cases, 23 countries, 48 species	30/01/2034
tritosulfuron		sulfonylurea herbicides	1 case, 1 country, 1 species	15/07/2025
pendimethalin	HRAC/WSSA 3	dinitroaniline herbicides	11 cases, 5 countries, 6 species	30/11/2024
propyzamide		amide herbicides	6 cases, 2 countries, 2 species	30/06/2025
2,4-D	HRAC/WSSA 4	phenoxy herbicides	47 cases, 16 countries, 25 species	31/12/2030
2,4-DB		phenoxy herbicides	not available	31/10/2032
aminopyralid		pyridinecarboxylic-acid herbicides	4 cases, 3 countries, 3 species	31/12/2024
clopyralid		pyridinecarboxylic-acid herbicides	4 case, 3 countries, 4 species	30/09/2036
dicamba		benzoic-acid herbicides	21 cases, 7 countries, 10 species	31/03/2027
dichlorprop-*P*		phenoxy herbicides	2 case, 1 country, 2 species	15/03/2025
florpyrauxifen-benzyl		pyridinecarboxylic-acid herbicides	2 cases, 2 species, 1 country	24/07/2029
fluoroxypyr		pyridyloxyacetic-acid herbicides	6 cases, 3 countries, 4 species	31/12/2024
halauxifen-methyl		pyridinecarboxylic-acid herbicides	not available	05/08/2025
MCPA		phenoxy herbicides	17 cases, 9 countries, 13 species	15/08/2026
MCPB		phenoxy herbicides	1 case, 1 country, 1 species	15/08/2026
mecoprop-*P*		phenoxy herbicides	3 case, 2 countries, 3 species	31/01/2024
picloram		pyridinecarboxylic-acid herbicides	5 cases, 3 countries, 5 species	15/02/2028
quinmerac		quinoline herbicides	not available	31/07/2024
triclopyr		pyridyloxyacetic-acid herbicides	1 case, 1 country, 1 species	15/12/2024
phenmedipham	HRAC/WSSA 5	bis-carbamate herbicides	1 case, 1 country, 1 species	15/02/2025
chlorotoluron		urea herbicides	16 cases, 7 countries, 6 species	15/08/2026
fluometuron		urea herbicides	not available	31/08/2024
lenacil		uracil herbicides	6 cases, 2 countries, 5 species	15/08/2025
metobromuron		urea herbicides	not available	31/12/2024
metribuzin		triazinone herbicides	30 cases, 11 countries, 16 species	15/02/2025
terbuthylazine		triazine herbicides	6 cases, 3 countries, 5 species	31/12/2024
bentazon	HRAC/WSSA 6	thiadiazine herbicides	3 cases, 2 countries, 3 species	31/05/2025
pyridate		diazine herbicides	not available	31/12/2030
glyphosate	HRAC/WSSA 9	organophosphorus herbicides	361 cases, 31 countries, 57 species	15/12/2033
beflubutamid	HRAC/WSSA 12	amide herbicides	not available	31/10/2026
diflufenican		anilide herbicides	7 cases, 2 countries, 4 species	15/01/2026
picolinafen		pyridinecarboxamide herbicides	not available	30/06/2031
fluorochloridone			not available	15/03/2026
clomazone	HRAC/WSSA 13	unclassified herbicides	3 cases, 2 countries, 3 species	15/06/2025
flumioxazin	HRAC/WSSA 14	dicarboximide herbicides	2 cases, 1 country, 2 species	28/02/2037
oxyfluorfen		diphenyl ether herbicides	3 cases, 3 countries, 3 species	31/12/2024
bifenox		diphenyl ether herbicides	not available	31/03/2027
carfentrazone-ethyl		triazolinone herbicides	5 cases, 4 countries, 4 species	31/07/2033
pyraflufen-ethyl		phenylpyrazole herbicides	2 cases, 1 country, 2 species	31/03/2031
dimethenamid-*P*	HRAC/WSSA 15	amide herbicides	1 case 1 country, 1 species	31/08/2034
dimethachlor		chloroacetanilide herbicides	not available	15/10/2026
ethofumesate		benzofurane herbicides	1 case, 1 country, 1 species	31/10/2031
flufenacet		anilide herbicides	6 cases, 4 countries, 2 species	15/06/2025
metazachlor		anilide herbicides	not available	31/10/2026
napropamide		amide herbicides	not available	31/03/2027
pethoxamid		amide herbicides	not available	30/11/2033
prosulfocarb		thiocarbamate herbicides	1 case, 1 country, 1 species	31/01/2027
triallate		thiocarbamate herbicides	12 cases, 3 countries, 2 species	31/03/2027
isoxaflutole	HRAC/WSSA 27	isoxazole herbicides	2 cases, 2 countries, 2 species	31/07/2034
mesotrione		triketone herbicides	16 cases, 3 countries, 4 species	31/05/2032
sulcotrione		triketone herbicides	not available	30/11/2026
tembotrione		triketone herbicides	10 cases, 2 countries, 3 species	31/07/2024
aclonifen	HRAC/WSSA 32	diphenyl ether herbicides	not available	31/10/2026

a The table reports the active principle (herbicide), the Mode of Action [MoA—according to the Herbicide Resistance Action Committee (HRAC) and the Weed Science Society of America (WSSA)], the harmonised FAO classification, the main herbicide resistance cases reported until 2024, and the expiration approval (Exp. Approval). The complete dataset detailing the herbicides (i.e., European nations in which they are allowed, WHO hazard class, etc.) is presented in Table S1. (https://ec.europa.eu/food/plant/pesticides/eu-pesticides-database/start/screen/active-substances^56^).

## 3 Overview of Herbicides Currently Registered in the EU

As of February 2024, the registry of authorized herbicides includes 82 active substances (Tables 2 and S1). This inventory, organized according to a harmonized classification of pesticides (Tables 2 and S1), reveals a diverse composition; e.g., phenoxy hormone products account for 7.3%, triazines for 2.4%, and carbamates, uracil, and dinitroaniline derivatives represent 1.2% each (with one active substance per category). Furthermore, urea derivatives comprise 3.7% of the list, while sulfonylureas, notable for their significant representation, constitute 18.3%. A broad category labeled “other herbicides” encompasses a heterogeneous collection of substances, making up 48.8% of the registry. These herbicides, according to acute risk to human health, are classified into three World Health Organization (WHO) hazard classes: 29% fall into class II, indicating moderate hazard; 31% into class III, denoting slight hazard; and a substantial 41% into class U, suggesting they are unlikely to pose an acute hazard under typical conditions of use. An example of the main different classes allowed in Europe, with a single molecule representing each specific class, is reported in Figure 1.

**Figure 1:**
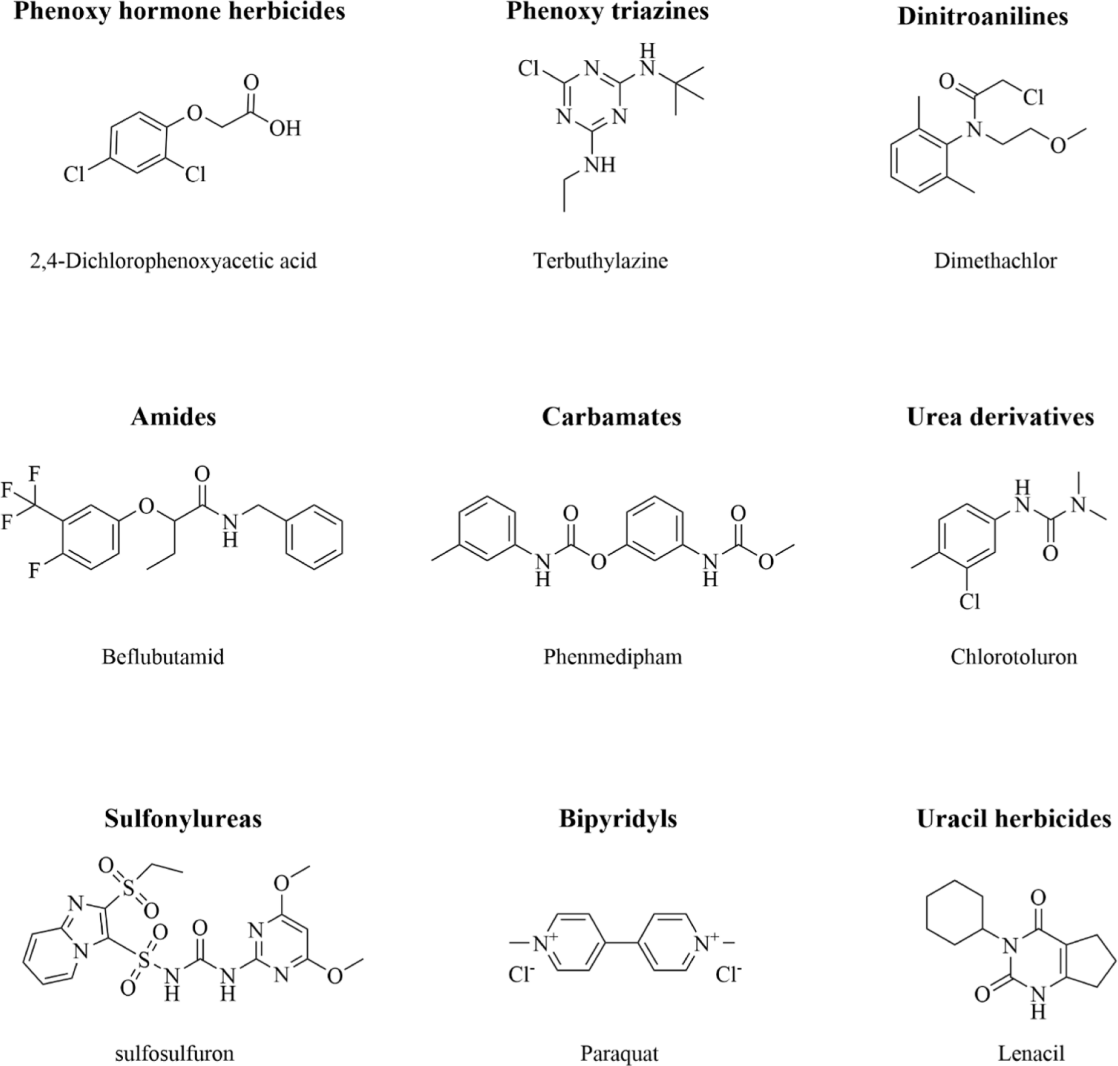
Chemical structures of representative herbicides from each of the nine main classes approved for use in Europe. Shown here are 2,4-dichlorophenoxyacetic acid as a prototypical phenoxy “hormone” herbicide, terbuthylazine as a phenoxy-triazine, and dimethachlor as a dinitroaniline. The amide class is exemplified by belflubenzamid, while phenmedipham illustrates the carbamates and chlorotoluron the urea derivatives. Sulfosulfuron represents the sulfonylureas, paraquat the bipyridyls, and lenacil one of the uracil-based herbicides. Together, these nine molecules typify the structural diversity and modes of action currently deployed in European weed-control programs.

### 3.1 Review of the Mode of Action (MoA) and Herbicide Resistance Status

Under the updated HRAC/WSSA classification, twenty-six distinct modes of action groups encompass 359 herbicides (Table 3). In the 2024 compilation of herbicides authorized within the European Union, merely 13 HRAC/WSSA groups are represented. Table 3 delineates the comprehensive inventory of herbicides, itemizing the total count alongside the subset registered within each HRAC/WSSA group. Additionally, it specifies the proportion of herbicides within each group accessible on the market in 2024, offering a clear overview of the current landscape in herbicide availability (Table 3).

**Table 3:** Number of Herbicides in Total and Registered in 2024 for Each HRAC/WSSA Group

mode of action	group number	total	registered in 2024	%
inhibition of acetyl CoA carboxylase	1	21	10	47.6
inhibition of acetolactate synthase	2	58	21	36.2
inhibition of microtubule assembly	3	18	2	11.1
auxin mimics	4	24	15	62.5
inhibition of photosynthesis at PSII - serine 264 binders	5	75	7	9.3
inhibition of enolpyruvyl shikimate phosphate synthase	9	1	1	100
inhibition of glutamine synthetase	10	2		
inhibition of phytoene desaturase	12	7	4	57.1
inhibition of deoxy-d-xylulose phosphate synthase	13	2	1	50
inhibition of protoporphyrinogen oxidase	14	30	5	16.7
inhibition of very long-chain fatty acid synthesis	15	43	9	20.9
inhibition of dihydropteroate synthase	18	1		
auxin transport inhibitor	19	2		
PS I electron diversion	22	4		
inhibition of microtubule organization	23	6		
uncouplers	24	6		
inhibition of hydroxyphenyl pyruvate dioxygenase	27	14	4	28.6
inhibition of dihydroorotate dehydrogenase	28	1		
inhibition of cellulose synthesis	29	6		
inhibition of fatty acid thioesterase	30	2		
inhibition of serine–threonine protein phosphatase	31	1		
inhibition of solanesyl diphosphate synthase	32	1		
inhibition of homogentisate solanesyltransferase	33	1		
inhibition of lycopene cyclase	34	1	1	100
auxin mimics/inhibition of cellulose synthesis	4/29	1		
inhibition of photosynthesis at PSII - histidine 215 binders/uncouplers	6/24	3	2	66.7
unknown		28		
in total		359	82	22.8

The predominant category of herbicides documented comprises inhibitors of acetolactate synthase (ALS), categorized under the HRAC/WSSA 2 group. This group consists of various chemical families, including sulfonylureas (15 herbicides), imidazolinones (1 herbicide), triazolinones (2 herbicides), and triazolopyrimidines (3 herbicides), collectively accounting for 26% of the active substances listed. ALS inhibitors are crucial for the biosynthesis of branched-chain amino acids such as valine, leucine, and isoleucine, impacting a wide range of plant species by impairing seedling growth. In mature plants, exposure may lead to various symptoms, including malformation, stunting, and diminished seed production. Remarkably, these herbicides exhibit such potency that they impact plant growth at levels below detectable standards of chemical analysis. The rapid emergence of resistance among weed species to ALS inhibitors is attributed to these compounds’ singular mode of action and extended residual activity.^68^ Notably, all 21 active substances within the HRAC/WSSA 2 group have been associated with selecting herbicide-resistant weeds.^64^ Resistance has developed across all substances listed, with iodosulfuron-methyl identified as the herbicide selected for resistance in the highest number of weed species—40 across 31 countries. Conversely, tritosulfuron has been linked to resistance in only one biotype of *Stellaria media* (L.) Vill. in Germany, despite its introduction in 2003 for postemergence application against dicotyledonous weeds in cereals and maize.^69,70^ Obviously, within each HRAC mode of action group, the fact that a herbicide has been more times linked to weed resistance cases or a specific weed has evolved more times resistance to herbicides depends on the use of this specific herbicide and the target weed, respectively. Perhaps sulfonylureas herbicides and, in particular, iodosulfuron-methyl^71^ are, after glyphosate, the most used herbicides in Europe, especially in cereals, as they can control a wide range of weeds,^72^ and forms part of numerous commercial herbicide mixtures. Therefore, the chance to find resistant weed species to iodosulfuron-methyl is higher than to other sulfonylurea herbicides.

The HRAC/WSSA 4 group of herbicides, comprising 15 active substances and accounting for 18% of the list, is the second most prevalent category. This group is chemically diverse, including phenoxy-carboxylic acids (with six herbicides) and pyridine carboxylic acids (with six herbicides as well), alongside three additional herbicides from varied chemical families: arylpicolinate (florpyrauxifen-benzyl), benzoic acids (dicamba), and quinoline carboxylic acids (quinmerac). Notably, this group spans several decades of herbicidal innovation. For instance, 2,4-D (2,4-dichlorophenoxyacetic acid) emerged as the inaugural member of this group, marking its global commercial release in 1945.^72^ Conversely, florpyrauxifen-benzyl represents a modern addition, securing EU approval in 2019. Renowned for its efficacy in postemergence applications, it effectively manages grasses, sedges, and broadleaf weeds in rice crops.^73^ Another recent development within this group is halauxifen-methyl (Arylex), formulated by Dow (Corteva Agriscience). This pyridine-type auxin-mimicking herbicide is remarkably potent against a spectrum of major broadleaf weeds, such as pigweed (*Amaranthus* spp.), henbit (*Lamium amplexicaule* L.), corn poppy (*Papaver rhoeas* L.), flixweed (*Descurainia sophia* (L.) Webb), and chickweed (*S. media*), at extremely low dosages of 5–10 g/ha, and is versatile across multiple crops, notably winter wheat.^73^ Despite the longstanding presence of auxin mimic herbicides on the market, nearly 70 years, global resistance cases are a total of 113, affecting 42 species. Of these, 25 species have demonstrated resistance to 2,4-D, indicating a relatively modest incidence of resistance across the globe.^64^

Within the list, ten graminicides, constituting 12% of the total, are categorized under the HRAC/WSSA 1 group as ACC-ase inhibitors. This group includes seven substances from the aryloxyphenoxypropionates chemical family (commonly referred to as FOPs), two from cyclohexanediones (DIMS), and one phenylpyrazole (DEN)—specifically, pinoxaden. Graminicides serve the purpose of controlling grassy weeds by inhibiting the activity of acetyl-CoA carboxylase (ACCase), a crucial enzyme in fatty acid biosynthesis. This blockage prevents the synthesis of lipids and secondary metabolites in susceptible plants, leading to compromised cell membrane integrity, leakage of metabolites, and eventual cell death. Notably, FOPs and DIMs were introduced to the agricultural market over four decades ago, while DEN entered the market more recently in 2006.^74^ Across all continents, each active substance within the HRAC/WSSA group 1 has been associated with the development of resistant weed species, with fenoxaprop-*P*-ethyl identified as the most prone herbicide to resistance selection.^64^ However, not all HRAC/WSSA group 1 herbicides induce the same mechanism of resistance to weeds. Some weed populations show metabolic resistance to some herbicides of this group, while they are sensitive to another active ingredients of the same group, and the same happens when target-site resistance is induced instead of metabolic resistance. This phenomenon is not exclusive of the HRAC/WSSA group 1 herbicides, it can also happen for HRAC/WSSA group 2, being the real problem when some weed populations show both types of resistance, target-site and metabolic.^75^

The fourth most prevalent category in the list pertains to the HRAC/WSSA group 15, which comprises nine active substances accounting for 11% of the total. These substances function by inhibiting very-long-chain fatty acid (VLCFA) elongases and have been employed for over 60 years for the residual control of weeds in a variety of crops including maize, barley, oat, sorghum, soybean, sugar cane, wheat, and certain vegetable crops. Their primary mechanism of action is the inhibition of shoot development in susceptible weed species, thereby preventing their emergence and growth.^76^ Three herbicides, dimethachlor, metazachlor, and pethoxamid, that have not been implicated in selecting resistant weed species are of special interest within this group. Pethoxamid is a relatively recent addition to the pesticide market, introduced in 2002. It is characterized as a pre- and early postemergence chloroacetamide herbicide targeting certain grasses and broad-leaved weeds in crops such as maize, soybeans, and sunflower. As a systemic herbicide, pethoxamid is absorbed by plants’ roots and young shoots^77^ Meanwhile, other herbicides in this group, such as Ethofumesate (1994) and prosulfocarb (2011), have been linked to a limited number of resistant weed cases, with flufenacet and triallate presenting more significant resistance challenges. These herbicides mostly target grass weeds, which have a greater ability to evolve herbicide detoxification mechanisms mediated by enhanced metabolic activity, and very few cases of cross-resistance.^78^

Seven herbicides are classified under the HRAC/WSSA 5 group and two under HRAC/WSSA 6, both encompassing photosynthesis inhibitors at photosystem II (PSII)—specifically, the serine 264 binders in group 5, and the histidine 215 binders in group 6. Historically, these groups were amalgamated under the HRAC classifications C1, C2, and C3, as general photosynthesis inhibitors at PSII. They have been delineated into groups 5 (comprising the former C1 and C2) and 6 (previously C3) due to the absence of demonstrated target site cross-resistance between these categories.^79^ This distinction underscores the indiscriminate nature of PSII targeting by these herbicides.^80^ Within HRAC/WSSA 5, the listed herbicides span several chemical classes including ureas (chlorotoluron, fluometuron, metobromuron), uracils (lenacil), triazines (terbuthylazine), triazinones (metribuzin), and phenyl-carbamates (phenmedipham). Meanwhile, HRAC/WSSA 6 features phenyl-pyridazines (pyridate) and benzothiadiazinones (bentazone). Both groups are selective, sparing crops while controlling weeds, and can be applied to either soil or foliage. Due to the abundance of binding sites in photosynthetic tissues, these herbicides can be used at higher rates. Upon exposure to full sunlight, treated plant leaves exhibit rapid wilting and death within days.^80^ Notably, fluometuron, metobromuron, and pyridate from these groups have not been linked to any weed resistance phenomena. Resistance is generally rare among these herbicides, with metribuzin and chlorotoluron recording the highest incidences of resistance cases, 30 and 16 respectively.

Group HRAC/WSSA 14 consists of five herbicides: carfentrazone-ethyl (*N*-phenyl triazolinones), flumioxazin (*N*-phenylphthalimides), pyraflufen-ethyl (phenylpyrazoles), oxyfluorfen, and bifenox (diphenylethers), which act by inhibiting protoporphyrinogen IX oxidase (Protox). This enzyme is crucial for converting protoporphyrinogen IX to protoporphyrin IX (Proto), with inhibition leading to uncontrolled substrate autoxidation and Proto accumulation. The resultant blockage of the porphyrin pathway halts chlorophyll synthesis, causing light-dependent damage (photobleaching) directly tied to Proto accumulation levels.^81^ These herbicides are approved for pre-emergence use to manage broadleaf weeds across various agricultural and horticultural settings. Among them, resistance occurrences are rare, with bifenox, introduced in the early 1970s, noting no confirmed resistance instances.^64^ Most of the herbicides in this group are contact herbicides. Oxyfluorfen is soil applied, and weeds die when trespassing on the soil surface when they try to emerge. On the contrary, carfentrazone and pyraflufen ethyl are foliar applied against dicot weeds, and they can also be used to desiccate potato leaves just after harvesting time.

In the classification of herbicides, HRAC/WSSA 12 designates phytoene desaturase inhibitors, represented by four herbicides: diflufenican and picolinafen (pyridinecarboxamides), beflubutamid (phenyl ethers), and fluorochloridone (pyrrolidine). These herbicides disrupt carotenoid biosynthesis in plants, leading to plant bleaching. Introduced commercially in the early 1970s, phytoene desaturase inhibitors comprise only a small fraction of the herbicides used in crop production.^82^ Beflubutamid, a newer addition to this class, was introduced in 2018.^83^ The resistance profile for these four herbicides is notable, with no resistance cases reported for picolinafen, fluorochloridone, and beflubutamid.^64^ Diflufenican, however, has been linked to seven cases of weed resistance.^84–86^

Following in sequence, HRAC/WSSA 27 encompasses inhibitors of *p*-hydroxyphenyl pyruvate dioxygenase (HPPD inhibitors) a group discovered through serendipitous observations of weed growth.^87^ HPPD inhibitors block the formation of homogentisic acid, an essential precursor for plastoquinone and vitamin E, making them highly effective for selective pre-emergence and, in certain cases, postemergence control of a broad spectrum of broadleaf and grass weeds in maize and rice. This efficacy has sparked interest in developing transgenic crops resistant to these herbicides.^88^ The group includes three triketones, mesotrione, sulcotrione, and tembotrione, and one isoxazole, isoxaflutole, with a relatively low incidence of weed resistance reported.^64^

In the group HRAC/WSSA 3, there are only two registered herbicides: propyzamide (chemical family benzamides) and pendimethalin (chemical family dinitroanilines). The HRAC/WSSA group 3 comprises herbicides that inhibit microtubule assembly by targeting tubulin proteins in plants and protists, and are called anticytoskeletal herbicides.^89^ They are used as pre-emergence herbicides to control various weed species. Both propyzamide and pendimethalin have been present on the pesticide market for decades; however, up-to-date, there are only 11 cases of herbicide resistance to pendimethalin and only six cases (2 countries and 2 species) to propyzamide.^64,90^

Glyphosate represents HRAC/WSSA group 9, inhibitors of enolpyruvyl shikimate phosphate synthase (EPSPS), an enzyme in the shikimate pathway that is essential for the biosynthesis of aromatic amino acids (phenylalanine, tryptophan, and tyrosine) in plants, fungi, microorganisms, and parasites.^91^ Glyphosate, a superior herbicide, was first commercialized in 1974, and became a highly successful nonselective herbicide before the introduction of glyphosate-tolerant crops.^92–94^ Since then, the use of glyphosate significantly increased, resulting in recent environmental, health, and safety risks.^95,96^ Until now, fifty-seven weed species in 31 countries have evolved resistance to this herbicide, with 361 cases of resistance.^64^ It is important to note that most of the glyphosate-resistant weeds appear in glyphosate-tolerant crops and fruit tree plantations with a massive and unique use of this active ingredient. Due to a massive use, glyphosate, endangers many nontarget organisms in the natural environment, comprising both soil and water.^97^ However, the glyphosate market aims for a positive forecast until 2035 ^97^ because it is a very effective herbicide, easy to manage, and inexpensive.

Clomazone, from the chemical family isoxazolidinones, is the only representative of HRAC/WSSA group 13, inhibitors of deoxy-d-xylulose phosphate synthase (DOXP). The DOXP is the first rate-limiting enzyme involved in the 2-*C*-methyl-d-erythritol 4-phosphate (MEP) pathway for terpenoid biosynthesis.^98^ Clomazone was developed in the early 1980s, a soil-applied, pre-emergence herbicide. It is used against broadleaf and grassy weeds. It is widely used for weed control in canopies of soybean, cotton, sugar cane, corn, rice, tobacco, and various vegetable crops. It is generally accepted that clomazone prevents the accumulation of chloroplast pigments and plastidic isoprene evolution.^99,100^ Despite a long clomazone’s presence on the market, it has only three cases of selected resistant weeds, one from Australia (1982) and two recent, 2008 and 2020, from the USA.^64^

Aclonifen is a unique diphenyl ether herbicide authorized for agronomic use in Europe in 1983. It is the only representative in the new group of solanesyl diphosphate synthase inhibitors HRAC/WSSA 32. Aclonifen is categorized as an inhibitor of pigment biosynthesis with an unknown target and is used agronomically for pre-emergence control of monocot and dicot weeds in potato, sunflower, lentils, and chickpea cultivation.^101^ Presently, there are no records for weed resistance to aclonifen.^64^

## 4 .Herbicides’ Use in Countries Representing European Biogeographic Regions

Agricultural production across European biogeographic regions directly correlates with pedoclimatic and agroeconomic conditions, such as diverse farm structures, ranging from numerous smallholdings in Southern and Eastern Europe to large, consolidated operations in Western and Northern Europe, which significantly influence herbicide usage patterns, including consumption rates from various FAO groups.^66^ Table 4 delineates these relationships, presenting the average herbicide usage per hectare of cropland. Notably, between 2016 and 2021, the Arctic bioregion reported the lowest herbicide application rates (Table 4). In contrast, the Atlantic bioregion experienced the highest levels of herbicide usage, closely followed by the Continental bioregion (Table 4). This variance underscores the impact of regional agricultural characteristics and environmental conditions on herbicide consumption trends.

**Table 4:** Agricultural Use of Herbicides in kg per Hectare of Cropland in 2016–2021 for European Bioregions Represented by Selected Countries

bioregions/country	2016	2017	2018	2019	2020	2021	mean
Atlantic/Belgium	2.588	2.717	3.028	2.651	2.195	2.761	2.657
Boreal/Estonia	0.864	0.674	0.621	0.733	0.484	0.861	0.706
Pannonian/Hungary	1.018	0.949	0.850	0.870	1.019	1.016	0.954
Arctic/Iceland	0.23	0.15	0.15	0.06	0.04	0.05	0.113
Mediterranean/Italy	0.792	0.746	0.751	0.914	1.040	0.587	0.805
Continental/Poland	1.133	1.209	1.001	1.025	1.111	1.253	1.122
Steppic/Romania	0.556	0.612	0.568	0.428	0.464	0.428	0.510
Alpine/Slovakia	0.612	0.646	0.665	0.674	0.674	0.644	0.653
Anatolian & Black Sea/Turkey	0.148	0.507	0.644	0.310	0.572	0.566	0.458

Table 5 illustrates trends in herbicide usage, which have shifted notably in recent years, displaying varying patterns across different countries that represent distinct biogeographical regions. This variability can be attributed to modifications in the European Union’s roster of registered herbicides, which has been progressively decreasing. Throughout the six years under review, each bioregion, denoted here as bioregion = country, experienced a reduction in herbicide use compared to the previous year on at least one occasion (Table 5). The Pannonian and Alpine regions exhibited a relatively stable pattern of herbicide application, with fluctuations of approximately 10% during the analyzed period. Conversely, other bioregions exhibited more pronounced variability in herbicide consumption. Notably, the Anatolian and Black Sea bioregions experienced a significant 236% increase in herbicide use in 2017 (Table 5).

**Table 5:** Year-to-Year Change (%) in Total Herbicide Use for Agricultural Purposes in 2016–2021 for European Bioregions Represented by Selected Countries (Previous Year = 100%)^a^

	change in herbicides use (%). previous year = 100%					
bioregion/country	2016	2017	2018	2019	2020	2021
Atlantic/Belgium	X	3.38	13.37	–11.75	–16.48	25.92
Boreal/Estonia	X	–23.40	–7.44	19.28	–33.76	79.50
Pannonian/Hungary	X	–6.78	–10.44	2.13	9.19	2.19
Arctic/Iceland	X	–33.33	0.00	–62.50	–40.00	31.11
Mediterranean/Italy	X	–4.98	–0.03	23.93	14.46	–43.73
Continental/Poland	X	7.59	–16.73	2.76	9.62	12.06
Steppic/Romania	X	9.59	–5.44	–22.65	2.81	–6.64
Alpine/Slovakia	X	5.27	3.30	1.43	–0.11	–5.87
Anatolian & Black Sea/Turkey	X	236.05	26.23	–52.04	85.09	0.18

a Source: own elaboration based on data from FAOSTAT^60^ and EUROSTAT.^61^

It is essential to acknowledge that variations in herbicide use are an inherent aspect of agricultural practices across various bioregions. The dominant crop types, such as cereals and root vegetables in the north and east, compared to high-value specialty crops like olives, grapes, and citrus in the south, require distinct weed management strategies and tailored herbicide selections. For instance, nonselective herbicides, such as glyphosate, are commonly used in orchards in the Mediterranean bioregion,^102,103^ where weed communities are composed of both perennial and annual species, and there is no tillage because it may increase soil erosion and reduce moisture retention. In contrast, selective herbicides are typically utilized for annual field crops with accompanying annual weed species, e.g., in the Continental and Atlantic bioregions. Furthermore, emerging technologies, such as precision weed control, offer promising solutions by enabling more targeted applications of herbicides, thereby reducing overall usage.^104^ This is particularly beneficial in bioregions with intensive agricultural production, such as those focused on vegetable crops.

Figures 1–10 offer an in-depth examination of the variation in herbicide application rates (expressed in kg per hectare of cropland) across various European bioregions and countries from 2016 to 2021. These analyses draw on data from FAOSTAT and employ the harmonized classification of herbicides, as detailed in Table 2, for those herbicides approved for use in 2024.

Phenoxy hormone herbicides, known for their complex action mechanisms similar to auxins (plant growth hormones), influence cellular division, enhance phosphate metabolism, and alter nucleic acid metabolism. Their primary application is controlling broadleaf weeds across various crops, including wheat, corn, and rice.^105^ Functioning as hormone mimics, these herbicides elevate the weed’s hormone levels beyond typical growth thresholds, thereby inhibiting weed growth and development, particularly in dicotyledonous species.^106^ From 2016 to 2021, the Continental and Atlantic bioregions reported the highest usage rates of phenoxy hormone herbicides, averaging 0.13 and 0.16 kg per hectare, respectively (Figure 2). Notably, their importance has declined in the Anatolian, Black Sea, and Steppic regions since 2019. Conversely, their application has remained relatively stable across other bioregions, with a notable increase of approximately 12% observed in the Continental region since 2019 (Figure 2).

**Figure 2:**
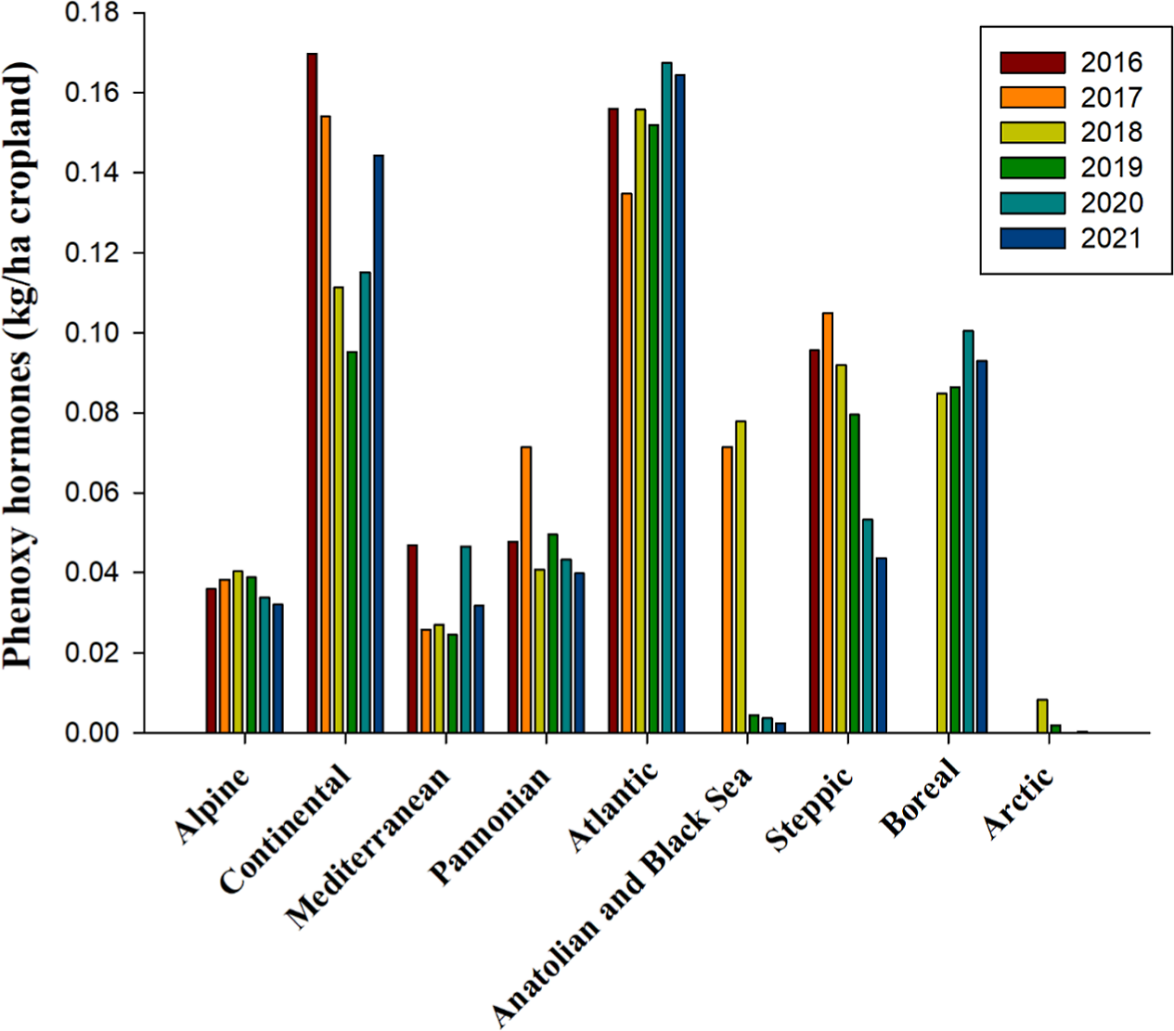
Use of phenoxy hormone herbicides, measured in kilograms per hectare of cropland from 2016 to 2021 across countries representing selected biogeographical regions of Europe. Notably, data for the Anatolian and Black Sea regions for 2016 and for the Boreal region for 2016 and 2017 are absent. This analysis is based on data meticulously compiled from FAOSTAT (https://www.fao.org)^60^ and Eurostat (https://ec.europa.eu/eurostat/data/database),^61^ representing a novel contribution by the authors. The bioregions under consideration are delineated by the following countries: Atlantic (Belgium), Boreal (Estonia), Pannonian (Hungary), Arctic (Iceland), Mediterranean (Italy), Continental (Poland), Steppic (Romania), Alpine (Slovakia), and Anatolian & Black Sea (Turkey). This geographic categorization allows for a comprehensive analysis of phenoxy hormone herbicide usage trends across diverse European agricultural landscapes.

As of 2024, the registry of approved herbicides lists only six phenoxy hormone herbicides, all categorized under the HRAC/WSSA group 4. Among these, one herbicide is set to expire by the end of January 2024, while the others are approved until 2026, 2030, or 2032 (Tables 2 and S1).

According to the Food and Agriculture Organization’s classification (WHO, 2022),^66^ triazines are defined by their structural composition of six-membered rings containing three nitrogen atoms. This structural feature, indicated by the prefix “tri-” for three and “azine” for a nitrogen-containing ring, characterizes the heterocyclic nature of triazines. As potent inhibitors of photosynthetic electron transport, triazine herbicides effectively restrict the oxidation of the quinone acceptor (QA), thereby impeding electron flow from photosystem II (PSII) to photosystem I (PSI) and ultimately inhibiting photosynthesis. This action results in a reduced maximum quantum yield, denoted as the Fv/Fm ratio.^107,108^ Furthermore, triazines compromise the turnover and stability of the D1 protein, essential for photosystem II’s repair mechanism, necessitating its replacement.^109^

Between 2016 and 2021, triazine herbicides were predominant in the Atlantic bioregion, with an average usage rate of 0.26 kg per hectare of cropland (Figure 3). Conversely, in other bioregions such as the Alpine, Mediterranean, and Steppic, their usage averaged below 0.05 kg per hectare. Notably, the Continental and Pannonian bioregions have observed an uptick in triazine usage since 2020, with rates approximately 70% lower than those in the Atlantic bioregion, averaging only 0.08 and 0.07 kg per hectare. In contrast, the Anatolian and Black Sea, Boreal, and Arctic bioregions have employed triazines only sparingly, with usage rates less than 0.004 kg per hectare or not at all (Figure 3). As of the 2024 registry, only two triazine herbicides, terbuthylazine and metribuzin, classified under HRAC/WSSA 5, remain authorized, with their approvals set to expire in December 2024 and February 2025, respectively (Tables 2 and S1).

**Figure 3:**
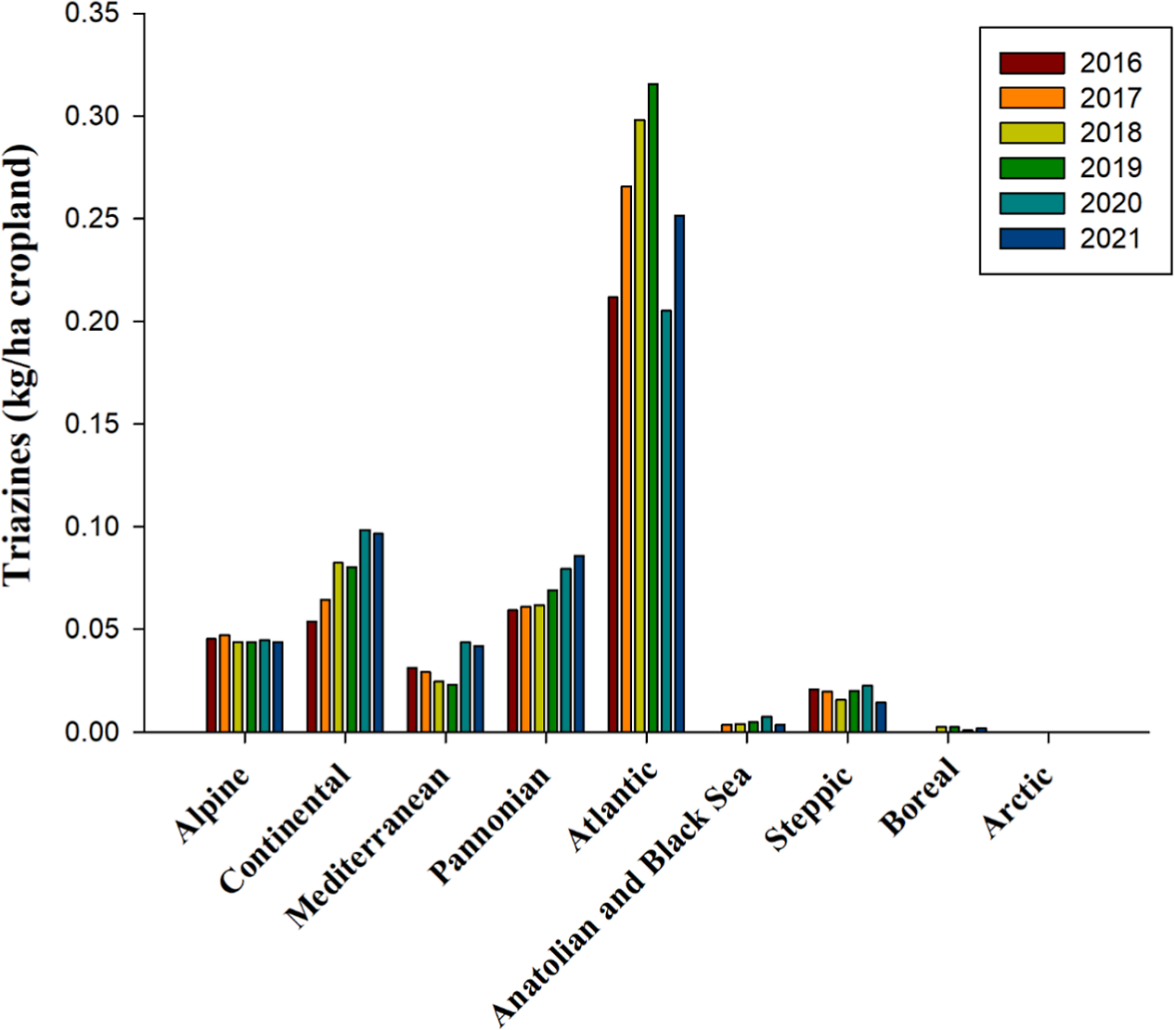
Use of phenoxy triazines, measured in kilograms per hectare of cropland from 2016 to 2021 across countries representing selected biogeographical regions of Europe. No data are available for Anatolian and Black Sea 2016 and Boreal 2016. Source and bioregions: As shown in Figure 1.

According to the FAO,^66^ amide herbicides exhibit many biological properties. Similar to triazine herbicides, those belonging to the amide group were notably more popular in the Atlantic bioregion than others. From 2016 to 2021, their average application rate in the Atlantic was 0.37 kg per hectare (Figure 4). This use significantly outpaced the Alpine and Continental bioregions, where amide herbicide application was approximately half of the Atlantic bioregion application. Moreover, it was roughly five to six times greater than in the Mediterranean and Pannonian bioregions. Notably, these herbicides saw scant use over the six years analyzed in the Anatolian, Black Sea, and Arctic bioregions (Figure 4). The 2024 registry of herbicides lists 12 substances categorized under amides and anilides, scheduled for approval expirations between 2025 and 2027 (Tables 2 and S1).

**Figure 4:**
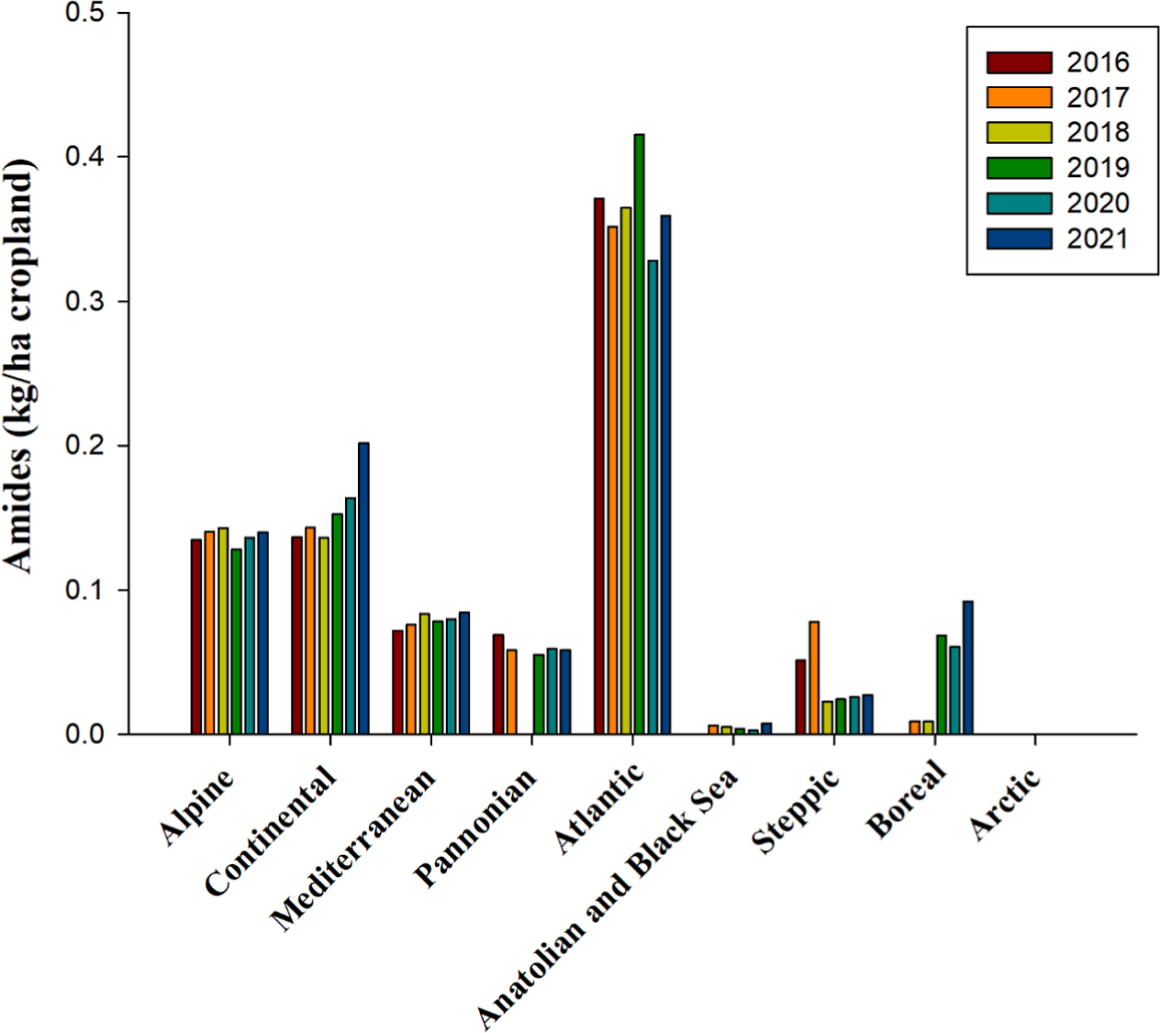
Use of amides, measured in kilograms per hectare of cropland, from 2016 to 2021 across countries representing selected biogeographical regions of Europe. No data is available for Anatolian & Black Sea 2016, Arctic 2017, Boreal 2016, and Pannonian 2018. Source and bioregions: like in Figure 1.

Carbamates, defined as carbamic acid esters,^110^ exhibited distinct use patterns across various European bioregions. In the Atlantic bioregion, carbamates were applied at the highest average rate of 0.13 kg per hectare of cropland, although this trend has declined in recent years (Figure 5). Conversely, there was an uptick in the use of carbamates in the Continental bioregion, in contrast to the Alpine bioregion, where their use noticeably decreased in 2021 (Figure 5). Between 2016 and 2021, the Alpine and Continental bioregions reported low average carbamate applications of 0.02 and 0.05 kg per hectare of cropland, respectively (Figure 5). Meanwhile, the Mediterranean region witnessed only marginal use of these herbicides, averaging 0.005 kg per hectare (Figure 5). As of the 2024 registry, carbamates in the list of authorized herbicides are solely represented by phenmedipham (classified under HRAC/WSSA group 5), which is set to expire in 2025 (Tables 2 and S1).

**Figure 5:**
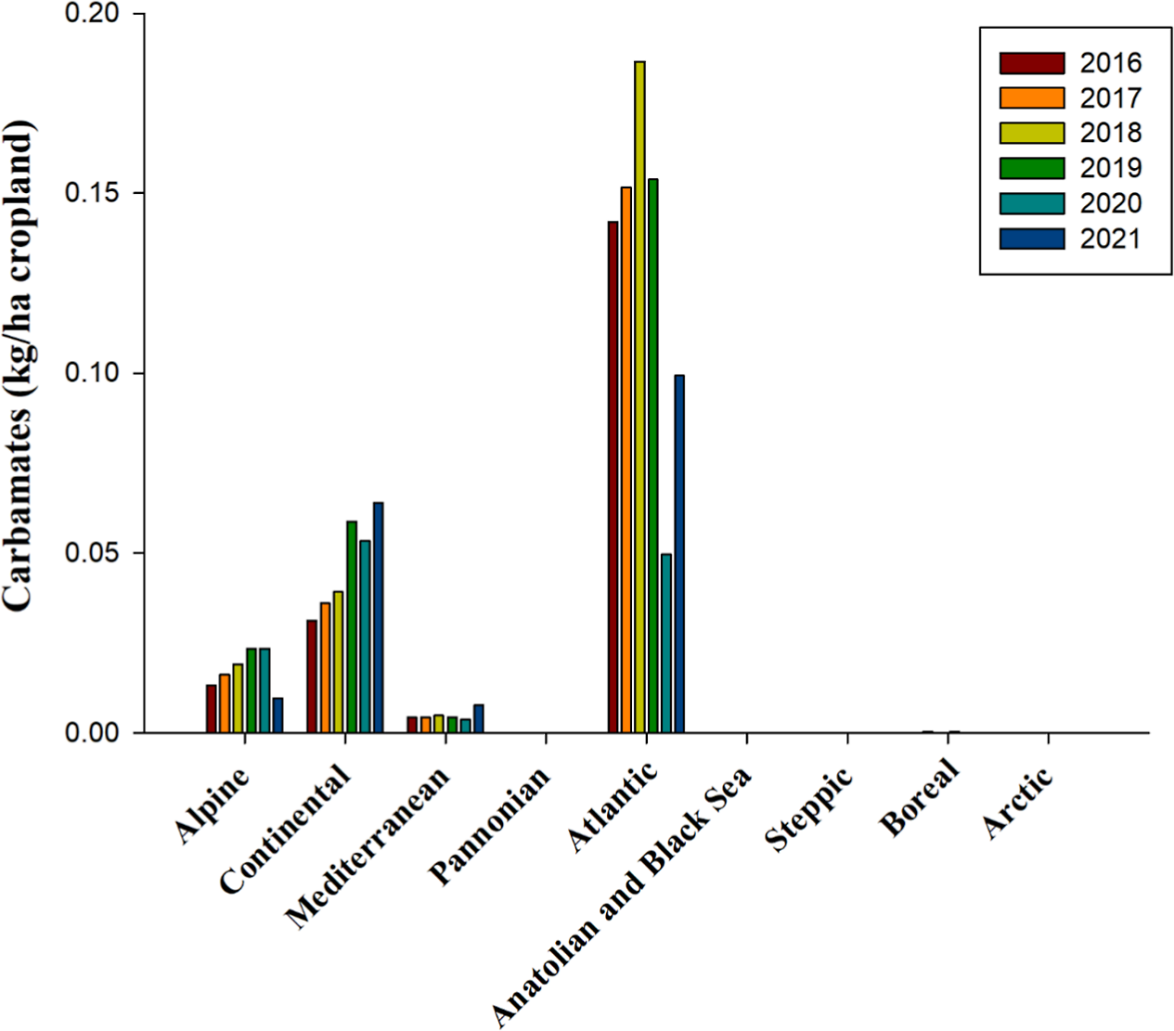
Use of carbamates, measured in kilograms per hectare of cropland, from 2016 to 2021 across countries representing selected biogeographical regions of Europe. No data is available for Anatolian & Black Sea 2016, 2018, 2021, Arctic 2016, 2017, 2018, Boreal 2016, and Pannonian 2016—source and bioregions, like in Figure 1.

Dinitroanilines, a chemical class derived from both aniline and dinitrobenzenes, function as antimitotic agents by disrupting microtubule polymerization and stability through their interaction with tubulin heterodimers.^111^ In European agriculture, spanning the biogeographical regions from 2016 to 2021, herbicides belonging to the dinitroaniline category were deployed in relatively modest quantities (Figure 6). Notably, the Atlantic bioregion reported the highest use, averaging 0.096 kg per hectare of cropland (Figure 6).

**Figure 6:**
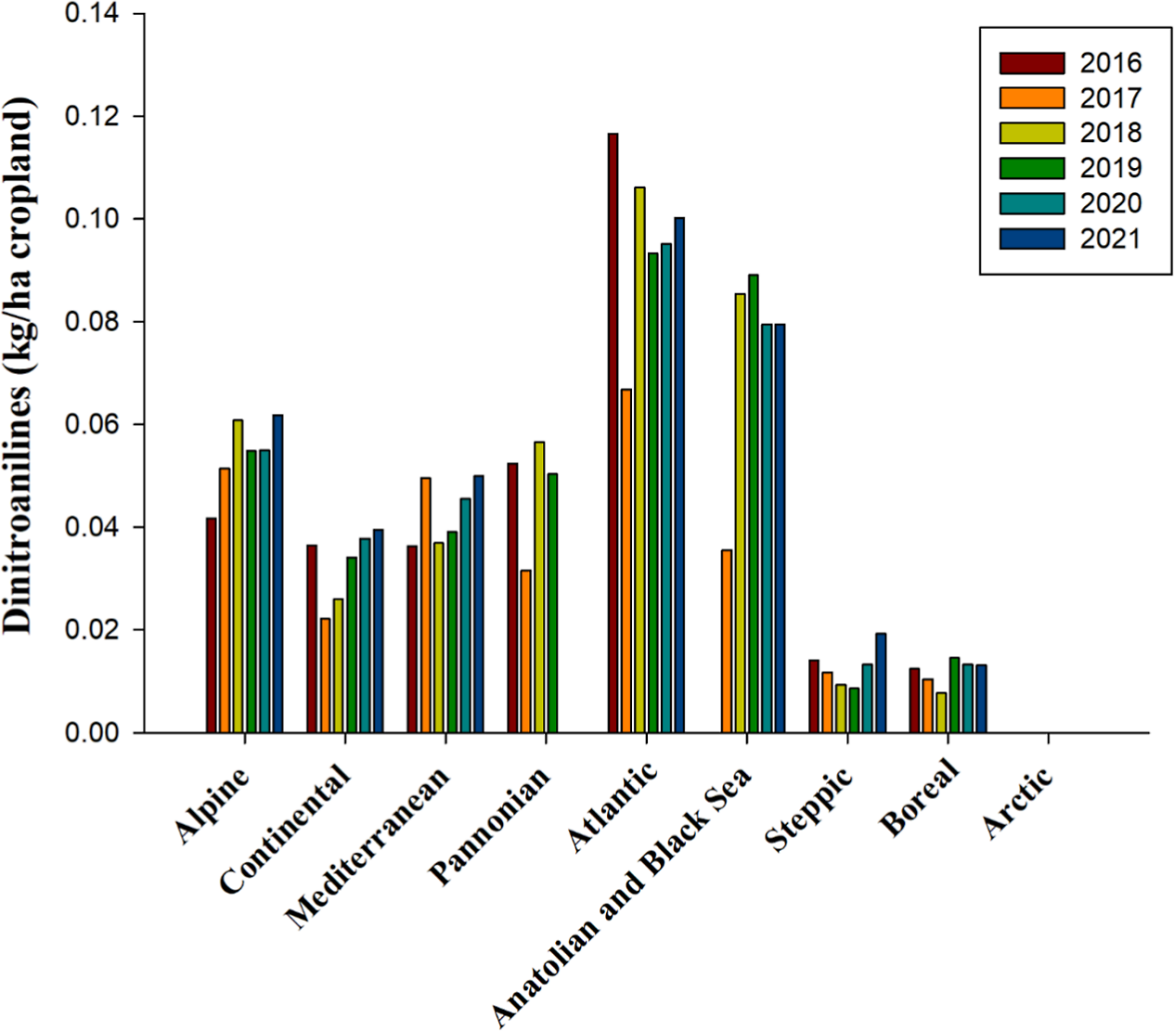
Use of dinitroanilines, measured in kilograms per hectare of cropland, over the years 2016 to 2021 across countries representing selected biogeographical regions of Europe. No data for Anatolian and Black Sea 2016, Arctic 2016, 2017, 2018, and Pannonian 2020, 2021 is available—source and bioregions, like in Figure 1.

A significant observation was made in 2017, where all bioregions, except the Mediterranean and Alpine regions, experienced a marked reduction in dinitroaniline application rates (Figure 6). However, after this decline, the use of these herbicides began to increase, eventually stabilizing through 2021 (Figure 6). The currently authorized herbicide registry lists pendimethalin as the sole dinitroaniline, slated for approval by the end of November 2024 (Tables 2 and S1). This trend underscores a cautious approach to dinitroaniline utilization across Europe, reflecting an adaptive management strategy in response to evolving agricultural and regulatory landscapes.

Urea derivatives experienced higher utilization within two specific bioregions—Atlantic and Continental—with discernible trends observed from 2016 to 2021 (Figure 7). In the Continental bioregion, use remained stable, averaging 0.09 kg per hectare of cropland. Conversely, the Atlantic bioregion witnessed a fluctuating pattern; after experiencing a decrease from 2017 to 2020, there was a notable resurgence of their use in 2021 (Figure 7). The average application rate of these herbicides in the Atlantic region over the six-year period was 0.12 kg per hectare of cropland (Figure 7). In comparison, use in other bioregions was relatively minimal, ranging from 0.001 to 0.02 kg per hectare of cropland (Figure 7). As of 2024, urea derivatives listed in the registry of herbicides are embodied by three specific compounds: chlorotoluron (expiration year: 2026), fluometuron (expiration year: 2024), and metobromuron (expiration year: 2024) (Tables 2 and S1). Chlortoluron (3-(3-chloro-*p*-tolyl)-1,1-dimethyl urea), developed by Ciba Geigy in 1969, is widely used to control grass weed in cereal, cotton, poppy and fruit crops.^112^ Fluometuron was introduced as a commercial chemical in 1960 by Ciba-Geigy AG under the trademark Cotoran; it has been widely used to control broadleaf weeds and grasses on agricultural crops e.g., cotton and sugar cane.^113^

**Figure 7:**
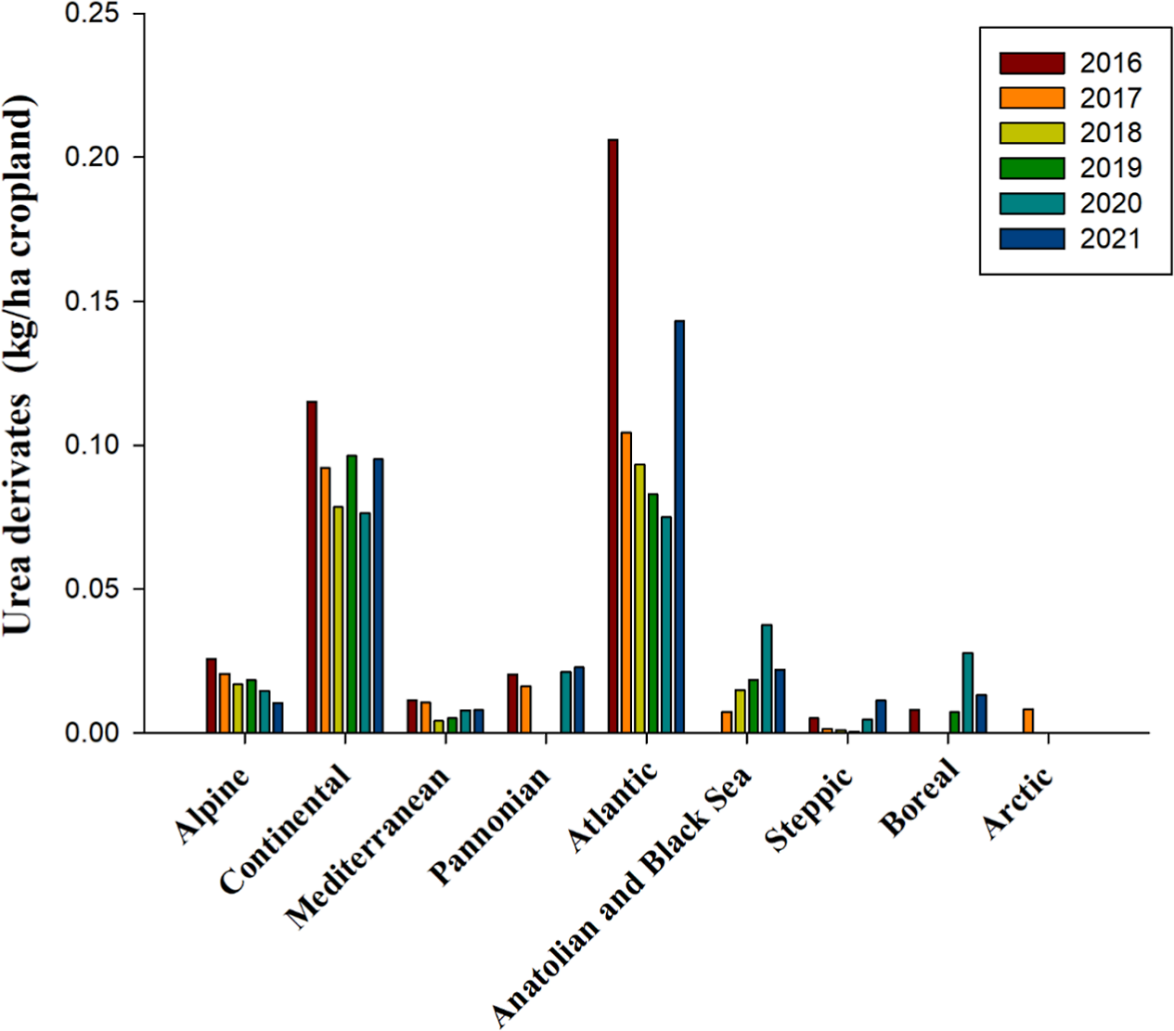
Use of urea derivatives, measured in kilograms per hectare of cropland, from 2016 to 2021 across countries representing selected biogeographical regions of Europe. No data for Anatolian & Black Sea 2016, Boreal 2017, 2018, and Pannonian 2018, 2019 is available—source and bioregions, like in Figure 1.

Sulfonylurea herbicides, known as meristematic inhibitors with foliar and soil activity, control broadleaf weeds more efficiently than grasses. They achieve this by disrupting the biosynthesis of the amino acids valine, isoleucine, and leucine, which are pivotal components in plant growth.^114^ Over the period from 2016 to 2021, sulfonylureas demonstrated a marked increase in significance within the Steppic bioregion, where their application averaged 0.022 kg per hectare of cropland (Figure 8). In contrast, their utilization across the Alpine, Continental, Mediterranean, and Atlantic bioregions remained consistent, averaging 0.006 to 0.013 kg per hectare during the same period (Figure 8). The FAOSTAT database indicates that the application of sulfonylurea herbicides was considerably less prevalent in the Arctic and Boreal bioregions, with uses ranging between 0.0001 and 0.002 kg per hectare of cropland (Figure 8).

**Figure 8:**
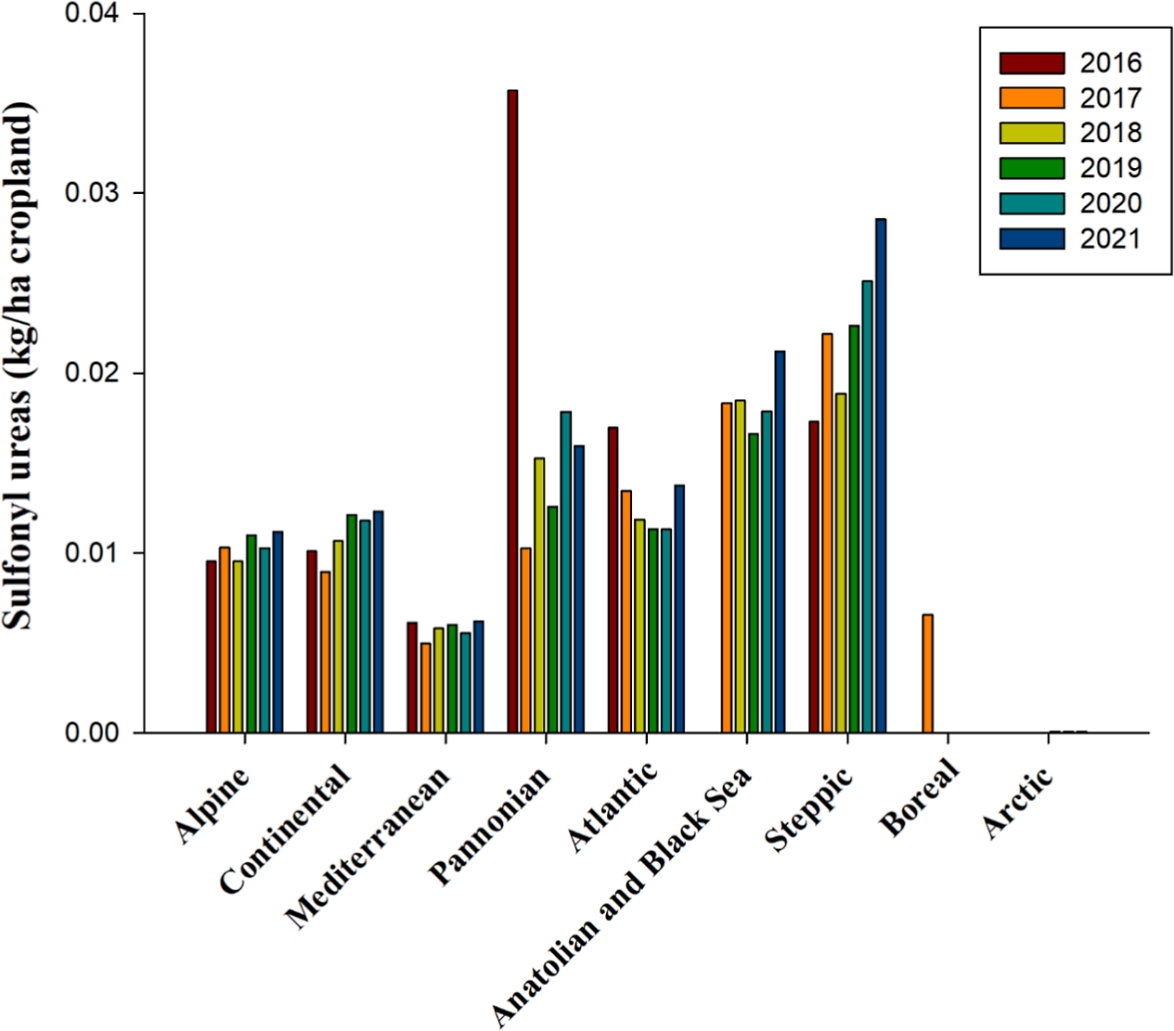
Use of sulfonylureas, measured in kilograms per hectare of cropland from 2016 to 2021 across countries representing selected biogeographical regions of Europe. No data is available for Anatolian & Black Sea 2016 and Boreal 2016, 2018—source and bioregions, like in Figure 1.

The 2024 herbicide registry lists 15 sulfonylurea compounds. Notably, two of these, metsulfuron-methyl and prosulfuron, are registered until 2024 (Tables 2 and S1). The remaining compounds, including Amidosulfuron, halosulfuron-methyl, and rimsulfuron, have expiration dates extending into 2025 and beyond, indicating a sustained reliance on and regulatory approval for using sulfonylurea herbicides in agricultural practices (Tables 2 and S1).

Bipyridyls constitute a family of chemical compounds characterized by two pyridyl rings. Pyridine, an aromatic nitrogen-containing heterocycle, can form complexes with most transition metals. The action mechanism of these herbicides involves positive ions, which are naturally dissociated and then reduced by photosynthesis to form stable free radicals. Subsequently, these free radicals are oxidized to regenerate the original ion and produce hydrogen peroxide, which destroys plant tissue.^114^ The application of bipyridyls has been notably significant in the Atlantic and Pannonian bioregions; however, there has been a marked decline since 2019 (Figure 9). Specifically, the average application rates of these herbicides were recorded at 0.05 kg per hectare of cropland in the Atlantic region and 0.03 kg per hectare in the Pannonian region, respectively (Figure 9). As of 2024, no bipyridyl herbicides are registered for use (Tables 2 and S1).

**Figure 9:**
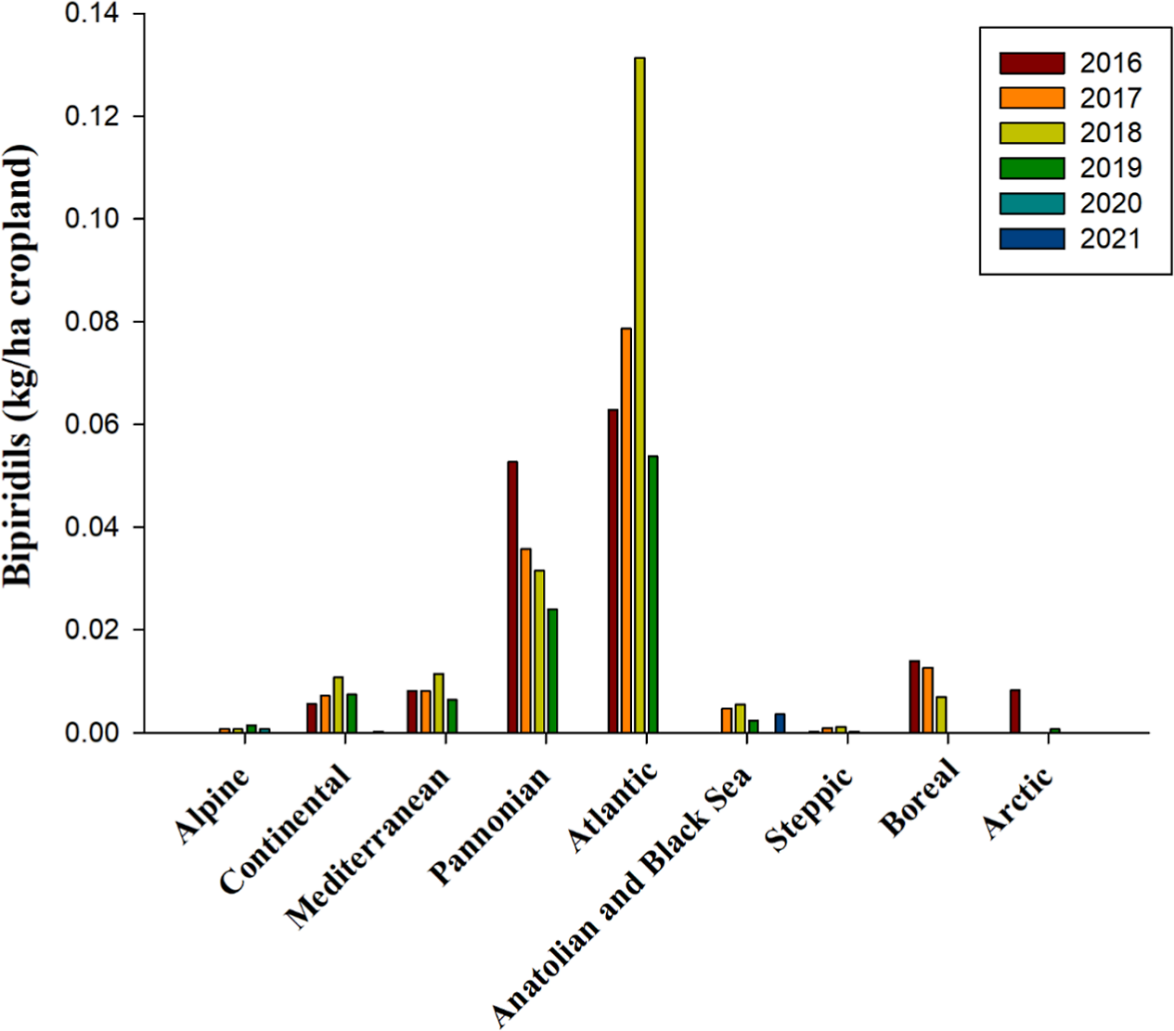
Bipyridyls, measured in kilograms per hectare of cropland, were used from 2016 to 2021 across countries representing selected biogeographical regions of Europe. No data is available for Anatolian & Black Sea 2016 and Pannonian 2021—source and bioregions, like in Figure 1.

Uracil herbicides decrease photosynthesis by inhibiting the Hill reaction, similarly to ureas and triazines. Typically applied to soil, they are absorbed and transported within plants via the transpiration stream.^114^ Notably, the Atlantic bioregion experienced a significant surge in the use of uracil herbicides, with a recorded increase of approximately 220% per hectare of cropland in 2021 compared to 2016 (Figure 10). The average application rates in the Alpine and Continental bioregions were 0.002 and 0.004 kg per hectare of cropland, respectively (Figure 10). Conversely, in the Mediterranean, Anatolian, Black Sea, and Steppic bioregions, the use of uracil herbicides was comparatively lower, averaging around 0.001 kg per hectare of cropland (Figure 10). Data on the use of this group of herbicides were largely unavailable for the Pannonian and Arctic bioregions for most of the years studied (Figure 10). As of 2024, lenacil stands as the sole uracil herbicide registered, with its approval set to expire in August 2025 (Tables 2 and S1).

**Figure 10:**
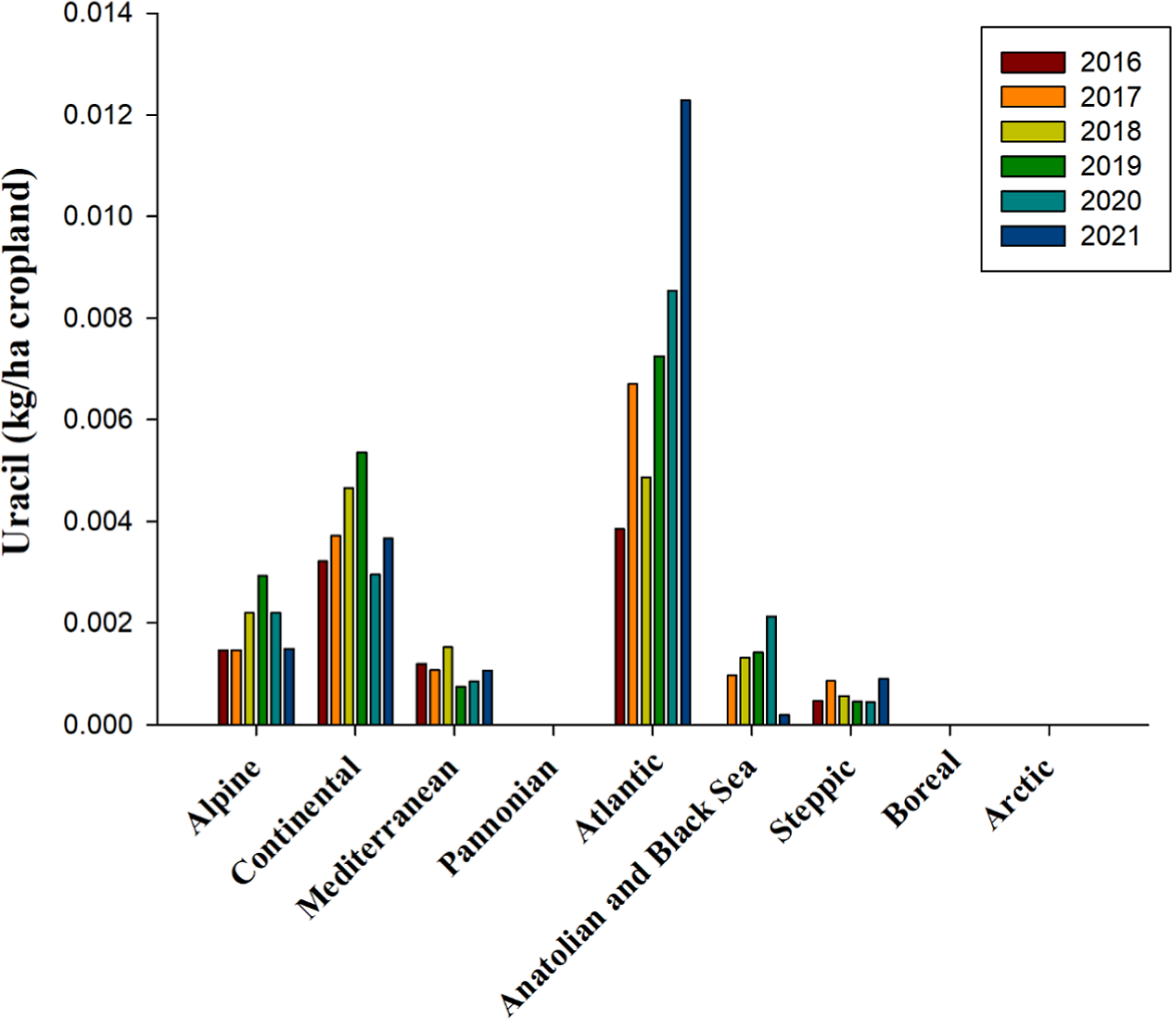
Use of uracil herbicides, measured in kilograms per hectare of cropland from 2016 to 2021 across countries representing selected biogeographical regions of Europe. No data is available for Anatolian & Black Sea 2016, Arctic 2016, 2017, 2018, Boreal 2016, and Pannonian 2016, 2017, 2018, and 2019—source and bioregions, like in Figure 1.

Within the FAO classification (https://www.fao.org),^66^ the “other herbicides” category encompasses diverse chemical classes, among which glyphosate’s significant contribution to agricultural productivity stands out. According to FAOSTAT data, this group’s herbicides have been deployed across all bioregions, with the Atlantic bioregion witnessing the most substantial utilization, averaging 1.46 kg per hectare of cropland, more than double the rate observed in other regions (Figure 11). From 2016 to 2021, the application of these herbicides remained relatively consistent, except in the Mediterranean bioregion, where a noticeable decline in their use was recorded in 2021 (Figure 11). The average application rates in other bioregions ranged from 0.33 to 0.59 kg per hectare, whereas the Arctic bioregion experienced minimal usage, with an application rate of only 0.006 kg per hectare (Figure 11). Notably, the 2024 registry of authorized herbicides identifies nearly half of the entries, 40 in total, as belonging to this “other” classification (Tables 2 and S1).

**Figure 11:**
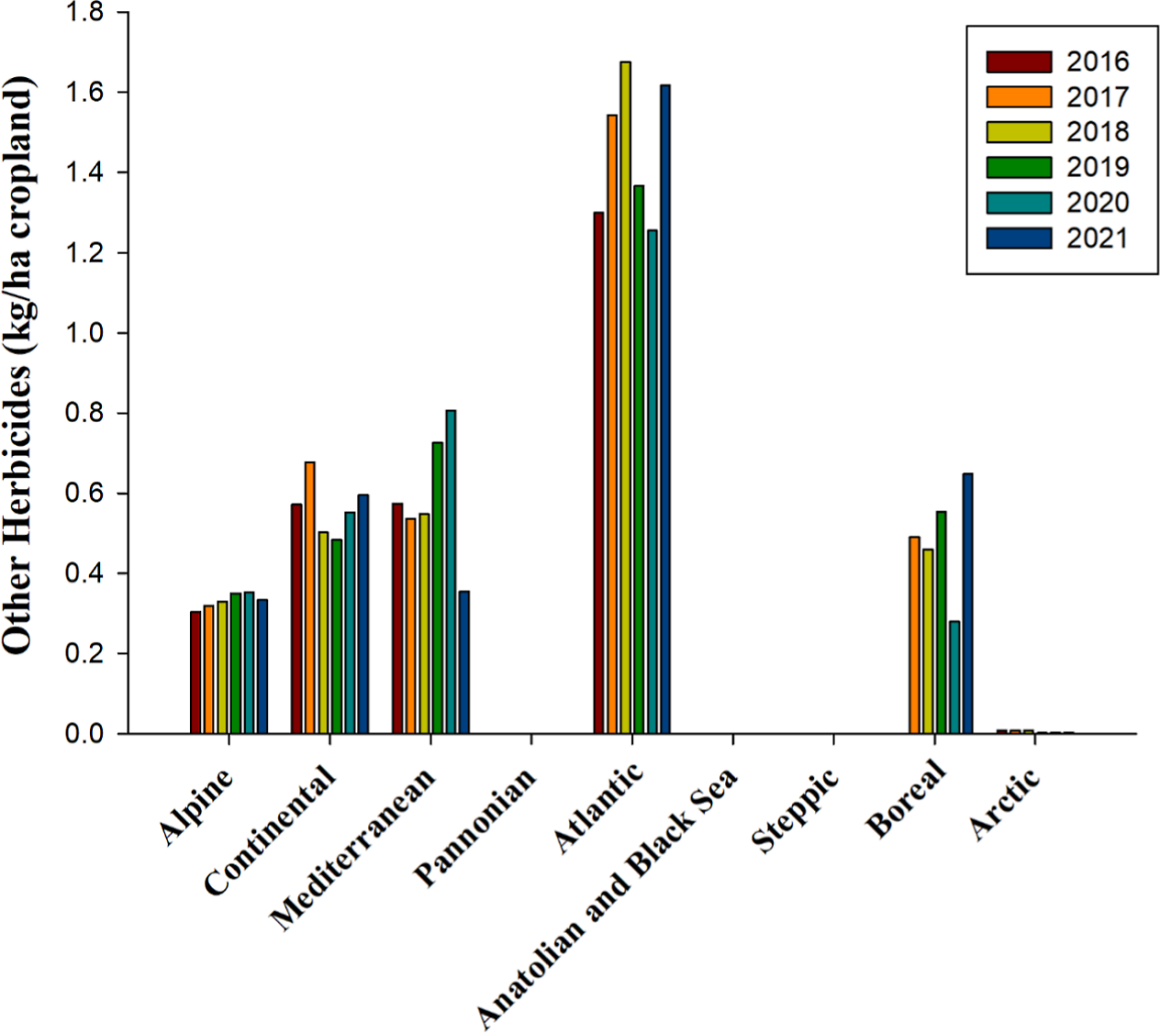
Other herbicides in kilograms per hectare of cropland used from 2016 to 2021 across countries representing selected biogeographical regions of Europe. Anatolian & Black Sea 2016, 2017, 2018, 2019, 2020, 2021, Boreal 2016, Pannonian 2016, 2017, 2018, 2019, 2020, 2021 and Steppic 2016, 2017, 2018, 2019, 2020, 2021—no data available. Source and bioregions: like in Figure 1.

Consolidating data on herbicide use across various European bioregions, as represented by selected countries from 2016 to 2021, reveals the consumption patterns of herbicides, measured in kilograms per hectare of cropland, with a predominant use within the Atlantic region across nearly all groups. The sole exception is observed with Sulfonylureas, where the average use per hectare of cropland surpassed that of the Atlantic region in the Steppic, Anatolian, Black Sea, and Pannonian bioregions.

Conversely, the Arctic bioregion exhibited markedly low or negligible herbicide usage, with instances where such data were absent from the FAOSTAT database for 2016–2021. Following the Atlantic, the Continental bioregion reported the next highest herbicide usage, succeeded by the Mediterranean region.

An annual summary of herbicide use per country from 2016 to 2021 was also analyzed and ranked in Table 6. This assessment indicated a shift in use proportions. Despite Poland’s smaller cropland area, representing the Continental bioregion, compared to Turkey’s (which represents the Anatolian and Black Sea bioregion), Poland emerged as the most prolific consumer of herbicides among the examined countries (Table 6). Romania, aligning with the Steppic bioregion, presents an intriguing case, with herbicide consumption nearly half that of Italy, despite both countries having comparable cropland areas (Table 6). Remarkably, Romania’s herbicide use aligns closely with Hungary’s, even though Hungary’s cropland area is half that of Romania’s (Table 6).

**Table 6:** Rank of Countries According to the Yearly Herbicide Use in Tons (Average for Years 2016-2021)

country	yearly herbicides used in tons	cropland area (ha)	rank
Poland	12,761,333	11,372,626	1
Turkey	10,659,205	23,325,833	2
Italy	7,462,666	9,267,057	3
Romania	4,611,728	9,056,166	4
Hungary	4,200,440	4,409,389	5
Belgium	2,330,013	877,373	6
Portugal	2,119,923	1,793,080	7
Slovakia	888,166	1,361,000	8
Estonia	491,881	696,000	9
Island	1465	12,902	10

## 5 Prospective Challenges and Opportunities

An in-depth review of herbicide use across Europe’s diverse agricultural landscapes reveals a multifaceted scenario characterized by the historical evolution of weed control methods, the progression toward herbicide-resistant crops, and the ensuing ecological and regulatory challenges.

This narrative illustrates the critical juncture at which modern agriculture finds itself, grappling with the dual imperatives of enhancing crop yields while preserving environmental integrity. The historical reliance on chemical herbicides, notably exemplified by the widespread adoption of herbicide-tolerant genetically modified crops (excluding those in Europe), has led to a notable reduction in herbicide diversity. This shift has inadvertently escalated the risks associated with herbicide resistance, with profound implications for biodiversity and the health of soil microbiota. Such developments underscore the inherent complexities within the European Union’s regulatory framework, which, despite its rigorous approach to herbicide approval and monitoring, has encountered significant controversies, most notably surrounding the use and regulation of glyphosate, still reregistered. These controversies underscore the need for independent, robust research to inform regulatory frameworks, ensuring they are based on comprehensive risk assessments and the latest scientific findings.

Moreover, the ecological impact of sustained herbicide use, as evidenced by the decline in biodiversity and the emergence of herbicide-resistant weed species, underscores the urgent need for sustainable weed management strategies. These strategies, which include reducing reliance on chemical herbicides in favor of crop rotation, biological control methods, and mechanical weeding, offer a pathway to mitigate the adverse impacts on ecosystems and human health. Such a shift necessitates reevaluating current regulatory policies and adopting policies that incentivize and support the transition toward more sustainable agricultural practices.

In response to these challenges, this analysis proposes a multifaceted approach that aligns with the objectives of the European Green Deal. Central to this strategy is the need for enhanced research into the long-term ecological impacts of herbicides, particularly their effects on soil health, nontarget species, and overall biodiversity. To ensure that policies remain effective and relevant, regulatory frameworks must evolve dynamically, incorporating the latest scientific findings, technological innovations, and the guiding principles of the Green Deal.

Specifically, policies should encourage the development and adoption of innovative weed management technologies and practices, thereby supporting the transition toward more sustainable agriculture. Practical measures, e.g., reducing the active dose of herbicide per unit area and sectorizing herbicide application, can significantly contribute to this goal.

At the European Union level, overarching policies, such as the Farm to Fork Strategy and the Common Agricultural Policy, set ambitious targets for pesticide reduction. Balancing agricultural productivity with environmental protection is a central objective of the European Green Deal, particularly within its Farm to Fork Strategy and the Common Agricultural Policy (CAP). The EU has established specific quantitative targets to guide this balance:

- Reduce pesticide use and risk by 50% by 2030
- Reduce nutrient losses by at least 50% by 2030, leading to a 20% reduction in fertilizer use
- Convert 25% of EU farmland to organic farming by 2030
- Reduce antimicrobial use in agriculture and aquaculture by 50% by 2030

These targets aim to enhance environmental sustainability while maintaining agricultural productivity. However, achieving them requires integrating environmental considerations into agricultural practices. The CAP plays a pivotal role by providing financial incentives for farmers to adopt sustainable practices, such as eco-schemes that reward environmentally friendly farming methods.

The European Union’s plan to reduce pesticide use by 50% by 2030, as part of the Green Deal’s Farm to Fork strategy, faced significant opposition from farmers across member states. In response to widespread protests and concerns about the potential impact on agricultural productivity and economic viability, the European Commission withdrew the proposal in early 2024. This decision was seen as a concession to farmers who argued that the proposed regulations would impose excessive burdens on their operations.

While the European Commission has indicated that the topic remains important, any future initiatives will likely focus on trade aspects and innovation rather than reinstating mandatory pesticide reduction targets.

However, implementation varies widely among member states due to factors such as national economic pressures, historical agricultural development, and public opinion. This divergence results in some countries increasing their use of herbicides while others achieve notable reductions.

Compounding these issues, the growing prevalence of herbicide resistance is driving a shift toward integrated weed management approaches, underscoring the need for continued adaptation and innovation in agricultural practices.

Moreover, there is a pronounced need for educational programs and technical support to guide farmers and agricultural stakeholders through the transition to sustainable weed management practices. These initiatives should promote the adoption of precision agriculture techniques to minimize herbicide use and maximize efficiency, thereby aligning agricultural productivity with environmental stewardship.

The European Community can significantly advance toward the sustainability goals outlined in the Green Deal by embracing integrated weed management practices, committing to ongoing research, and implementing forward-thinking policies. This comprehensive strategy addresses the immediate challenges of herbicide resistance and ecological degradation. It sets the stage for a more sustainable and resilient agricultural future, harmonizing the dual objectives of enhancing food security and preserving the natural environment.

The widespread use of glyphosate, a highly effective and economically vital herbicide for modern farming, exemplifies a significant geopolitical and agronomic conflict. While glyphosate remains widely authorized and used in Europe due to its practicality and low cost, it faces increasing public and governmental scrutiny, with growing pressure for restrictions. Despite its effectiveness, extensive use has contributed to the development of resistance in weeds, now documented in 60 species across 31 countries, and has caused detrimental effects on nontarget organisms, including those in soil and aquatic ecosystems. These environmental and safety concerns underscore the urgent need for independent, robust research to better understand glyphosate’s long-term impacts. To mitigate these risks, the adoption of sustainable alternatives and more judicious use of glyphosate are strongly recommended.

## Supplementary Material



## Notes

Supplementary Table S1: The Supporting Information (Table S1) provides a comprehensive inventory of all 82 herbicidal active substances authorized for use in the European Community as of 2024. For each entry, the table lists the common name and molecular formula, the Harmonized FAO classification (group and subgroup), the HRAC/WSSA mode-of-action code, the number of documented resistance cases worldwide (Heap 2024), the WHO hazard class, the EU approval and expiration dates, and the member states in which the substance is currently authorized (PDF)
