# Supplementary material for: Herbicides in Use: Current Status and Perspectives in the Different Biogeographic Regions of Europe
Source: J Agric Food Chem. 2025 Aug 26;73(36):22089–109. doi: 10.1021/acs.jafc.5c03867 (PMC12426928; doi:10.1021/acs.jafc.5c03867)
Supplement: Supplementary file 1 [file jf5c03867_si_001.pdf]

SUPPLEMENTARY INFORMATION

Herbicides in Use: Current Status and Perspectives in the Different Biogeographic Regions of Europe

Agnieszka Synowiec[1*], Marta Czekaj[2], Mercedes Verdeguer[3], Diego G. De Barreda Ferraz[3], Claudia Campillo-Cora[4,5], Yedra Vieites-Álvarez[5,6], David López-González[5,6], Adela M. Sánchez-Moreiras[5,6], David Fernández-Calviño[4,5], Fabio F. Nocito[7], Carla Ragonezi[8], Miguel A. Almeida Pinheiro de Carvalho[8], Merit Sutri[9], Merrit Shanskiy[9], Sigrún Dögg Eddudóttir[10], Tatiana P. Fedoniuk[11], Andrea Vityi[12], Ursula Bürgener[13], Liliana Piron[14], Mihai Gidea[14], Francisco Espinosa Escrig[15], Gülçin Beker Akbulut[16], Alicia Morugán Coronado[17], Esther Valiño[18], Fabrizio Araniti[7*]

Supplementary Table S1: The Supporting Information (Table S1) provides a comprehensive inventory of all 82 herbicidal active substances authorised for use in the European Community as of 2024. For each entry, the table lists the common name and molecular formula, the Harmonized FAO classification (group and subgroup), the HRAC/WSSA mode-of-action code, the number of documented resistance cases worldwide (Heap 2024), the WHO hazard class, the EU approval and expiration dates, and the member states in which the substance is currently authorized.

Herbicide	Harmonized FAO classification	Harmonized FAO classification	Molecular formula	MoA	Herbicide resistance cases [Heap 2024]	WHO hazard class	date of approval	expiration of approval	Authorised countries
Fenoxaprop-P-ethyl	other	Aryloxyphenoxy-propionic herbicides	C16H12ClNO5	HRAC/WSSA 1	122 cases, 35 countries, 26 species	III	01/01/2009	15/08/2025	AT, BE, BG, CY, CZ, DE, DK, EE, EL, ES, FI, FR, FR, FR, HR, HU, IE, LT, LU, LV, NL, PL, PT, RO, SE, SK, UK
Fluazifop-P-butyl	other	Aryloxyphenoxy-propionic herbicides	C15H12F3NO4	HRAC/WSSA 1	55 cases, 16 countries, 23 species	III	01/01/2012	31/05/2026	AT, BE, BG, CY, CZ, DE, EE, EL, ES, FI, FR, FR, FR, HR, HU, IE, IT, LT, LU, LV, NL, PL, PT, RO, SE, SI, SK, UK
Propaquizafop	other	Aryloxyphenoxy-propionic herbicides	C22H22ClN3O5	HRAC/WSSA 1	13 cases, 9 countries, 10 species	U	01/12/2009	28/02/2027	AT, BE, BG, CY, CZ, DE, DK, EE, EL, ES, FI, FR, FR, FR, HR, HU, IE, LT, LU, LV, PL, PT, RO, SE, SI, SK, UK
Quizalofop-P-ethyl	other	Aryloxyphenoxy-propionic herbicides	C19H17ClN2O4	HRAC/WSSA 1	40 cases, 15 countries, 20 species	II	01/12/2009	28/02/2027	AT, BE, BG, CY, CZ, DE, EE, EL, ES, FI, FR, FR, FR, HR, HU, IE, LT, LU, LV, MT, NL, PL, PT, RO, SE, SI, SK, UK
Clodinafop	other	Aryloxyphenoxy-propionic herbicides	C14H11ClFNO4	HRAC/WSSA 1	78 cases, 25 countries, 13 species		01/02/2007	15/12/2025	BG, CY, DE, DK, EL, ES, FR, FR, FR, HR, IE, NL, PL, PT, RO, UK
Cyhalofop-butyl	other	Aryloxyphenoxy-propionic herbicides	C20H20FNO4	HRAC/WSSA 1	25 cases, 13 countries, 9 species	U	01/07/2017	30/06/2032	BG, EL, ES, FR, FR, FR, PT
Diclofop	other	Aryloxyphenoxy-propionic herbicides	C15H12Cl2O4	HRAC/WSSA 1	87 cases, 20 countries, 13 species	II	01/06/2011	31/08/2026	EL, ES, PT
Clethodim	other	Cyclohexanedione herbicides	C17H26ClNO3S	HRAC/WSSA 1	34 cases, 12 countries, 16 species		01/06/2011	31/08/2026	AT, BE, BG, CY, CZ, DE, EE, EL, ES, FI, FR, FR, FR, HR, HU, IE, IT, LT, LU, LV, NL, PL, PT, RO, SE, SI, SK, UK
Cycloxydim	other	Cyclohexanedione herbicides	C17H27NO3S	HRAC/WSSA 1	26 cases, 10 countries, 9 species	III	01/06/2011	31/08/2026	AT, BE, BG, CY, CZ, DE, DK, EE, EL, ES, FI, FR, FR, FR, HR, HU, IE, LT, LU, LV, NL, PL, PT, RO, SE, SI, SK, UK
Pinoxaden	other	Phenylpyrazole herbicides	C23H32N2O4	HRAC/WSSA 1	60 cases, 18 countries, 16 species	III	01/07/2016	30/06/2026	AT, BE, BG, CY, CZ, DE, EE, EL, ES, FI, FR, FR, FR, HR, HU, IE, IT, LT, LU, LV, NL, PL, PT, RO, SE, SI, SK, UK
Florasulam	amides and anilides	Anilide herbicides	C12H8F3N5O3S	HRAC/WSSA 2	36 cases, 17 countries, 23 species	U	01/01/2016	31/12/2030	AT, BE, BG, CY, CZ, DE, DK, EE, EL, ES, FI, FR, FR, FR, HR, HU, IE, IT, LT, LU, LV, MT, NL, PL, PT, RO, SE, SI, SK, UK
Penoxsulam	amides and anilides	Amide herbicides	C16H14F5N5O5S	HRAC/WSSA 2	29 cases, 13 countries, 14 species	U	01/08/2010	15/05/2026	AT, BE, CY, CZ, DE, EL, ES, FR, FR, FR, HU, LU, PT, RO, SI, SK
Pyroxsulam	amides and anilides	Amide herbicides	C14H13F3N6O5S	HRAC/WSSA 2	56 cases, 19 countries, 27 species	III	01/05/2014	30/04/2025	AT, BE, BG, CY, CZ, DE, DK, EE, EL, ES, FI, FR, FR, FR, HR, HU, IE, LT, LU, LV, NL, PL, PT, RO, SE, SI, SK, UK
Imazamox	other	Imidazolinone herbicides	C15H19N3O4	HRAC/WSSA 2	74 cases, 18 countries, 43 species	III	01/11/2017	31/01/2025	AT, BE, BG, CY, CZ, DE, EE, EL, ES, FI, FR, FR, FR, HR, HU, IE, LT, LV, MT, NL, PL, PT, RO, SE, SI, SK, UK

Propoxycarbazone	other	Triazolone herbicides	C15H18N4O7S	HRAC/WSSA 2	18 cases, 10 countries, 12 species		01/09/2017	31/08/2032	BE, BG, CY, CZ, DE, EE, EL, ES, FI, FR, FR, FR, IE, LU, LV, PL, PT, RO, SE, SK, UK
Thiencarbazone-methyl	other	Triazolone herbicides	C12H14N4O7S2	HRAC/WSSA 2	6 cases, 4 countries, 5 species		01/07/2014	30/09/2024	AT, BE, BG, CY, CZ, DE, DK, EE, EL, ES, FI, FR, FR, FR, HR, HU, IE, LT, LU, LV, NL, PL, PT, RO, SE, SI, SK, UK
Amidosulfuron	sulfonylurea herbicides	Sulfonylurea herbicides	C9H15N5O7S2	HRAC/WSSA 2	5 cases, 5 countries, 5 species		01/01/2009	15/08/2025	AT, BE, BG, CZ, DE, EE, EL, ES, FI, FR, FR, FR, HR, HU, IE, IT, LT, LU, LV, NL, PL, PT, RO, SE, SI, SK, UK
Bensulfuron	sulfonylurea herbicides	Sulfonylurea herbicides	C15H16N4O7S	HRAC/WSSA 2	58 cases, 13 countries, 31 species	U		15/08/2026	AT, BG, CY, EL, FR, FR, FR, HR, HU, IT, MT, NL, PL, PT, RO, SI
Flazasulfuron	sulfonylurea herbicides	Sulfonylurea herbicides	C13H12F3N5O5S	HRAC/WSSA 2	5 cases, 3 countries, 4 species	III	01/08/2017	31/07/2032	AT, BE, BG, CY, CZ, DE, EL, ES, FR, FR, FR, HR, HU, IE, LU, MT, PL, PT, RO, SK, UK
Foramsulfuron	sulfonylurea herbicides	Sulfonylurea herbicides	C17H20N6O7S	HRAC/WSSA 2	27 cases, 11 countries, 16 species		01/06/2020	31/05/2035	AT, BE, BG, CY, CZ, DE, DK, EE, EL, ES, FI, FR, FR, FR, HR, HU, IE, IT, LT, LU, LV, NL, PL, PT, RO, SE, SI, SK, UK
Halosulfuron -methyl	sulfonylurea herbicides	Sulfonylurea herbicides	C13H15ClN6O7S	HRAC/WSSA 2	25 cases, 5 countries, 20 species			05/08/2025	BG, EL, FR, FR, FR, HU, PT
Iodosulfuron-methyl	sulfonylurea herbicides	Sulfonylurea herbicides	C14H14IN5O6S	HRAC/WSSA 2	118 cases, 31 countries, 40 species			31/03/2032	AT, BE, BG, CY, CZ, DE, DK, EE, EL, ES, FI, FR, FR, FR, HR, HU, IE, LT, LU, LV, NL, PL, PT, RO, SE, SI, SK, UK
Mesosulfuron-methyl	sulfonylurea herbicides	Sulfonylurea herbicides	C17H21N5O9S2	HRAC/WSSA 2	89 cases, 24 countries, 26 species			30/06/2032	AT, BE, BG, CY, CZ, DE, DK, EE, EL, ES, FI, FR, FR, FR, HR, HU, IE, IT, LT, LU, LV, NL, PL, PT, RO, SE, SI, SK, UK
Metsulfuron-methyl	sulfonylurea herbicides	Sulfonylurea herbicides	C14H15N5O6S	HRAC/WSSA 2	84 cases, 18 countries, 40 species	U	01/04/2016	31/03/2024	AT, BE, BG, CY, CZ, DE, DK, EE, EL, ES, FI, FR, FR, FR, HR, HU, IE, LT, LU, LV, MT, NL, PL, PT, RO, SE, SI, SK, UK
Nicosulfuron	sulfonylurea herbicides	Sulfonylurea herbicides	C15H18N6O6S	HRAC/WSSA 2	59 cases, 18 countries, 27 species	U	01/01/2009	31/03/2027	AT, BE, BG, CY, CZ, DE, EE, EL, ES, FR, FR, FR, HR, HU, IE, LT, LU, LV, MT, NL, PL, PT, RO, SI, SK, UK
Prosulfuron	sulfonylurea herbicides	Sulfonylurea herbicides	C15H16F3N5O4S	HRAC/WSSA 2	8 cases, 4 countries, 7 species		01/05/2017	31/07/2024	AT, BE, BG, CZ, DE, EL, ES, FR, FR, FR, HR, HU, IT, LU, MT, NL, PL, PT, RO, SI, SK, UK
Rimsulfuron (aka renriduron)	sulfonylurea herbicides	Sulfonylurea herbicides	C14H17N5O7S2	HRAC/WSSA 2	17 cases, 9 countries, 12 species	U	01/02/2007	15/08/2025	AT, BE, BG, CY, CZ, DE, EE, EL, ES, FI, FR, FR, FR, HR, HU, IE, LT, LU, LV, MT, NL, PL, PT, RO, SE, SI, SK, UK
Sulfosulfuron	sulfonylurea herbicides	Sulfonylurea herbicides	C16H18N6O7S2	HRAC/WSSA 2	22 cases, 12 countries, 14 species		01/01/2016	31/12/2030	BE, CZ, EE, FI, FR, FR, FR, HU, IE, IT, LV, SK, UK
Thifensulfuron-methyl	sulfonylurea herbicides	Sulfonylurea herbicides	C12H13N5O6S2	HRAC/WSSA 2	93 cases, 13 countries, 31 species	U	01/11/2016	31/10/2031	AT, BE, BG, CZ, DE, DK, EE, EL, ES, FI, FR, FR, FR, HR, HU, IE, LT, LU, LV, NL, PL, PT, RO, SE, SI, SK, UK
Tribenuron (aka metometuron)	sulfonylurea herbicides	Sulfonylurea herbicides	C14H15N5O6S	HRAC/WSSA 2	105 cases, 23 countries, 48 species	U		30/01/2034	AT, BE, BG, CY, CZ, DE, DK, EE, EL, ES, FI, FR, FR, FR, HR, HU, IE, LU, LV, MT, NL, PL, PT, RO, SE, SI, SK, UK
Tritosulfuron	sulfonylurea herbicides	Sulfonylurea herbicides	C13H9F6N5O4S	HRAC/WSSA 2	1 case, 1 country, 1 species		01/12/2008	15/07/2025	AT, BE, CZ, DE, DK, EL, ES, FI, FR, FR, FR, HR, HU, IE, NL, PL, RO, SE, SK, UK
Propyzamide	amides and anilides	Amide herbicides	C12H11Cl2NO	HRAC/WSSA 3	6 cases, 2 countries, 2 species	U	01/07/2018	30/06/2025	AT, BE, CY, CZ, DE, DK, EL, FR, FR, FR, HU, IE, IT, LU, MT, NL, PL, PT, SE, UK

Pendimethalin	dinitroaniline derivatives	Dinitroaniline herbicides	C13H19N3O4	HRAC/WSSA 3	11 cases, 5 countries, 6 species	II	01/09/2017	30/11/2024	AT, BE, BG, CY, CZ, DE, DK, EE, EL, ES, FI, FR, FR, FR, HR, HU, IE, LT, LU, LV, MT, NL, PL, PT, RO, SI, SK, UK
Florpyrauxifen-benzyl	other	Pyridinecarboxylic-acid herbicides	C20H14Cl2F2N2O3	HRAC/WSSA 4	2 cases, 2 species, 1 country		24/07/2019	24/07/2029	BG, EL, ES, FR, FR, FR
Dicamba	other	Benzoic-acid herbicides	C8H6Cl2O3	HRAC/WSSA 4	21 cases, 7 countries, 10 species	II	01/01/2009	31/03/2027	AT, BE, BG, CY, CZ, DE, DK, EE, EL, ES, FI, FR, FR, FR, HR, HU, IE, LT, LU, LV, MT, NL, PL, PT, RO, SI, SK, UK
Aminopyralid	other	Pyridinecarboxylic-acid herbicides	C6H4Cl2N2O2	HRAC/WSSA 4	4 cases, 3 countries, 3 species	U	01/01/2015	31/12/2024	AT, BE, BG, CZ, DE, DK, EE, EL, ES, FI, FR, FR, FR, HR, HU, IE, IT, LT, LU, LV, NL, PL, PT, RO, SE, SI, SK, UK
Clopyralid	other	Pyridinecarboxylic-acid herbicides	C6H3Cl2NO2	HRAC/WSSA 4	4 case, 3 countries, 4 species	III	01/10/2021	30/09/2036	AT, BE, BG, CY, CZ, DE, DK, EE, EL, ES, FI, FR, FR, FR, HR, HU, IE, IT, LT, LU, LV, NL, PL, PT, RO, SE, SI, SK, UK
Fluroxypyr	other	Pyridyloxyacetic-acid herbicides	C7H5Cl2FN2O3	HRAC/WSSA 4	6 cases, 3 countries, 4 species	U	01/01/2012	31/12/2024	AT, BE, BG, CY, CZ, DE, DK, EE, EL, ES, FI, FR, FR, FR, HR, HU, IE, LT, LU, LV, MT, NL, PL, PT, RO, SE, SI, SK, UK
Halauxifen-methyl	other	Pyridinecarboxylic-acid herbicides	C14H11Cl2FN2O3	HRAC/WSSA 4	*Not available*		05/08/2015	05/08/2025	AT, BE, BG, CY, CZ, DE, DK, EE, FI, FR, FR, FR, HU, IE, LT, LU, LV, NL, PL, PT, RO, SE, SI, SK, UK
Picloram	other	Pyridinecarboxylic-acid herbicides	C6H3Cl3N2O2	HRAC/WSSA 4	5 cases, 3 countries, 5 species	U	01/01/2009	15/02/2028	AT, BG, CZ, DE, DK, EE, ES, FI, FR, FR, FR, HR, HU, IE, LT, LU, LV, PL, RO, SE, SI, SK, UK
Triclopyr	other	Pyridyloxyacetic-acid herbicides	C7H4Cl3NO3	HRAC/WSSA 4	1 case, 1 country, 1 species	II	01/06/2007	15/12/2024	AT, BE, CY, CZ, DE, EL, ES, FR, FR, FR, IE, LU, NL, PL, PT, RO, SK, UK
Quinmerac	other	Quinoline herbicides	C11H8ClNO2	HRAC/WSSA 4	*Not available*	U	01/05/2011	31/07/2024	AT, BE, BG, CZ, DE, EE, EL, FI, FR, FR, FR, HR, HU, IE, LT, LU, LV, NL, PL, RO, SE, SI, SK, UK
2,4-D	phenoxyphytohormones	Phenoxy herbicides	C8H6Cl2O3	HRAC/WSSA 4	47 cases, 16 countries, 25 species	II	01/01/2016	31/12/2030	AT, BE, BG, CY, CZ, DE, DK, EE, EL, ES, FI, FR, FR, FR, HR, HU, IE, IT, LT, LU, LV, MT, NL, PL, PT, RO, SE, SI, SK, UK
2,4-DB	phenoxyphytohormones	Phenoxy herbicides	C10H10Cl2O3	HRAC/WSSA 4	*Not available*	II	01/11/2017	31/10/2032	ES, IE, NL, UK
Dichlorprop-P	phenoxyphytohormones	Phenoxy herbicides	C9H8Cl2O3	HRAC/WSSA 4	2 case, 1 country, 2 species	II	01/06/2007	15/03/2025	
MCPA	phenoxyphytohormones	Phenoxy herbicides	C9H9ClO3	HRAC/WSSA 4	17 cases, 9 countries, 13 species	II	01/05/2006	15/08/2026	AT, BE, BG, CY, CZ, DE, DK, EE, EL, ES, FI, FR, FR, FR, HR, HU, IE, LT, LU, LV, MT, NL, PL, PT, RO, SE, SI, SK, UK
MCPB	phenoxyphytohormones	Phenoxy herbicides	C11H13ClO3	HRAC/WSSA 4	1 case, 1 country, 1 species	II	01/05/2006	15/08/2026	AT, BE, CZ, EE, FR, FR, FR, HU, IE, LT, LU, LV, PL, RO, SK, UK
Mecoprop-P	phenoxyphytohormones	Phenoxy herbicides	C10H11ClO3	HRAC/WSSA 4	3 case, 2 countries, 3 species	II	01/06/2004	31/01/2024	AT, BE, BG, CY, CZ, DE, EE, EL, ES, FI, FR, FR, FR, HR, HU, IE, LT, LU, LV, NL, PL, PT, SI, SK, UK
Phenmedipham	carbamates	Bis-carbamate herbicides	C16H16N2O4	HRAC/WSSA 5	1 case, 1 country, 1 species	U	01/03/2005	15/02/2025	AT, BE, BG, CY, CZ, DE, DK, EE, EL, ES, FI, FR, FR, FR, HR, HU, IE, LT, LU, LV, NL, PL, PT, RO, SE, SK, UK
Terbuthylazine	triazine	Triazine herbicides	C9H16ClN5	HRAC/WSSA 5	6 cases, 3 countries, 5 species	III	01/01/2012	31/12/2024	AT, BE, BG, CY, CZ, DE, EL, ES, FR, FR, FR, HR, HU, IE, IT, LU, MT, NL, PL, PT, RO, SI, SK, UK
Metribuzin	triazine	Triazinone herbicides	C8H14N4OS	HRAC/WSSA 5	30 cases, 11 countries, 16 species	II	01/10/2007	15/02/2025	AT, BE, BG, CY, CZ, DE, EE, EL, ES, FI, FR, FR, FR, HR, HU, IE, IT, LU, LV, MT, NL, PL, PT, RO, SE, SI, SK, UK
Lenacil	uracil	Uracil herbicides	C13H18N2O2	HRAC/WSSA 5	6 cases, 2 countries, 5 species	U	01/01/2009	15/08/2025	AT, BE, CY, CZ, DE, EL, ES, FR, FR, FR, HU, IE, NL, PL, PT, RO, SK, UK

Chlorotoluron	urea	Urea herbicides	C10H13ClN2O	HRAC/WSSA 5	16 cases, 7 countries, 6 species	U	01/03/2006	15/08/2026	
Fluometuron	urea	Urea herbicides	C10H11F3N2O	HRAC/WSSA 5	*Not available*	U	01/06/2011	31/08/2024	
Metobromuron	urea	Urea herbicides	C9H11BrN2O2	HRAC/WSSA 5	*Not available*	III	01/01/2015	31/12/2024	AT, BE, BG, CY, CZ, DE, DK, EE, EL, ES, FI, FR, FR, FR, HR, HU, IE, LT, LU, LV, MT, NL, PL, PT, RO, SE, SI, SK, UK
Bentazon	other	Thiadiazine herbicides	C10H12N2O3S	HRAC/WSSA 6	3 cases, 2 countries, 3 species	II	01/06/2018	31/05/2025	BE, BG, CY, CZ, DK, EE, EL, ES, FI, FR, FR, FR, HR, HU, IE, IT, LT, LU, LV, NL, PL, PT, RO, SE, SI, SK, UK
Pyridate	other	Diazine herbicides	C19H23ClN2O2S	HRAC/WSSA 6	*Not available*	III	01/01/2016	31/12/2030	AT, BE, BG, CY, CZ, DE, DK, EE, EL, ES, FI, FR, FR, FR, HR, HU, IE, LT, LU, LV, MT, NL, PL, PT, RO, SE, SI, SK, UK
Glyphosate	other	Organophosphorus herbicides	C3H8NO5P	HRAC/WSSA 9	361 cases, 31 countries, 57 species	III	16/12/2017	15/12/2033	AT, BE, BG, CY, CZ, DE, DK, EE, EL, ES, FI, FR, FR, FR, HR, HU, IE, LT, LV, MT, NL, PL, PT, RO, SE, SI, SK, UK
Beflubutamid	amides and anilides	Amide herbicides	C18H17F4NO2	HRAC/WSSA 12	*Not available*		01/12/2007	31/10/2026	BE, BG, CZ, DE, EL, ES, FR, FR, FR, IT, LU, PL, RO
Diflufenican	amides and anilides	Anilide herbicides	C19H11F5N2O2	HRAC/WSSA 12	7 cases, 2 countries, 4 species	III	01/01/2009	15/01/2026	AT, BE, BG, CY, CZ, DE, DK, EE, EL, ES, FI, FR, FR, FR, HR, HU, IE, LT, LU, LV, MT, NL, PL, PT, RO, SE, SI, SK, UK
Picolinafen	other	Pyridinecarboxamide herbicides	C19H12F4N2O2	HRAC/WSSA 12	*Not available*		01/11/2016	30/06/2031	AT, BE, BG, CZ, DE, DK, EE, ES, FR, FR, FR, HU, IE, IT, LT, LU, LV, PL, SE, UK
Flurochloridone			C12H10Cl2F3NO	HRAC/WSSA 12	*Not available*		01/06/2011	15/03/2026	CZ, EL, FR, FR, FR, HR, HU, IT, PL, RO, SK
Clomazone	other	Unclassified herbicides	C12H14ClNO2	HRAC/WSSA 13	3 cases, 2 countries, 3 species	II	01/11/2008	15/06/2025	AT, BE, BG, CY, CZ, DE, DK, EE, EL, ES, FR, FR, FR, HR, HU, IE, LT, LU, LV, MT, NL, PL, PT, RO, SE, SI, SK, UK
Oxyfluorfen	other	Diphenyl ether herbicides	C15H11ClF3NO4	HRAC/WSSA 14	3 cases, 3 countries, 3 species	U	01/01/2012	31/12/2024	BG, CY, EL, ES, HR, MT, PL, PT, RO
Bifenox	other	Diphenyl ether herbicides	C14H9Cl2NO5	HRAC/WSSA 14	*Not available*	U	01/01/2009	31/03/2027	AT, BE, BG, CZ, DE, EE, ES, FI, FR, FR, FR, HU, IT, LT, LU, LV, NL, PL, RO, SE, SK, UK
Flumioxazin	other	Dicarboximide herbicides	C19H15FN2O4	HRAC/WSSA 14	2 cases, 1 country, 2 species	III	01/03/2022	28/02/2037	AT, BE, BG, CZ, DE, EL, ES, HR, HU, IE, LV, NL, RO, UK
Pyraflufen-ethyl	other	Phenylpyrazole herbicides	C15H13Cl2F3N2O4	HRAC/WSSA 14	2 cases, 1 country, 2 species		01/04/2016	31/03/2031	AT, BE, BG, CZ, DE, EL, ES, FR, FR, FR, HU, IE, LU, NL, PL, PT, RO, UK
Carfentrazone-ethyl	other	Triazolinone herbicides	C15H14Cl2F3N3O3	HRAC/WSSA 14	5 cases, 4 countries, 4 species		01/08/2018	31/07/2033	AT, BE, CZ, DE, EE, EL, ES, FI, FR, FR, FR, HU, IE, IT, LT, LU, LV, MT, NL, PL, PT, SE, SK, UK
Dimethachlor	amides and anilides	Chloroacetanilide herbicides	C13H18ClNO2	HRAC/WSSA 15	*Not available*		01/01/2010	15/10/2026	AT, BG, CZ, DE, EE, FR, FR, FR, HR, HU, IE, LT, LV, PL, RO, SI, SK, UK
Dimethenamid-P	amides and anilides	Amide herbicides	C12H18ClNO2S	HRAC/WSSA 15	1 case 1 country, 1 species	II	01/09/2019	31/08/2034	AT, BE, BG, CY, CZ, DE, EL, ES, FI, FR, FR, FR, HR, HU, IE, LT, LU, LV, NL, PL, PT, RO, SE, SI, SK, UK
Metazachlor	amides and anilides	Anilide herbicides	C14H16ClN3O	HRAC/WSSA 15	*Not available*	III	01/08/2009	31/10/2026	AT, BE, BG, CY, CZ, DE, EE, EL, ES, FI, FR, FR, FR, HR, HU, IE, IT, LT, LU, LV, MT, NL, PL, PT, RO, SI, SK, UK
Pethoxamid	amides and anilides	Amide herbicides	C16H22ClNO2	HRAC/WSSA 15	*Not available*		01/12/2018	30/11/2033	AT, BE, BG, CZ, DE, EL, ES, FR, FR, FR, HR, HU, LU, PL, PT, RO, SI, SK
Napropamide	amides and anilides	Amide herbicides	C17H21NO2	HRAC/WSSA 15	*Not available*	U	01/01/2011	31/03/2027	AT, BE, BG, CZ, DE, EE, EL, ES, FI, FR, FR, FR, HR, HU, IE, LT, LU, LV, NL, PL, RO, SE, SI, SK, UK
Flufenacet	amides and anilides	Anilide herbicides	C14H13F4N3O2S	HRAC/WSSA 15	6 cases, 4 countries, 2 species	II	01/01/2004	15/06/2025	AT, BE, BG, CY, CZ, DE, EE, EL, ES, FR, FR, FR, HR, HU, IE, LT, LU, LV, MT, NL, PL, PT, RO, SI, SK, UK

Ethofumesate	other	Benzofurane herbicides	C13H18O5S	HRAC/WSSA 15	1 case, 1 country, 1 species	U	01/11/2016	31/10/2031	AT, BE, BG, CZ, DE, DK, EL, ES, FI, FR, FR, FR, HR, HU, IE, LT, LU, LV, NL, PL, PT, RO, SE, SK, UK
Prosulfocarb	other	Thiocarbamate herbicides	C14H21NOS	HRAC/WSSA 15	1 case, 1 country, 1 species	II	01/11/2009	31/01/2027	AT, BE, BG, CY, CZ, DE, DK, EE, EL, ES, FI, FR, FR, FR, HR, HU, IE, LT, LU, LV, NL, PL, PT, RO, SE, SI, SK, UK
Tri-allate	other	Thiocarbamate herbicides	C10H16Cl3NOS	HRAC/WSSA 15	12 cases, 3 countries, 2 species	III	01/01/2010	31/03/2027	BE, FR, FR, FR, IE, NL, UK
Isoxaflutole	other	Isoxazole herbicides	C15H12F3NO4S	HRAC/WSSA 27	2 cases, 2 countries, 2 species	III	01/08/2019	31/07/2034	AT, BE, BG, CZ, DE, EL, ES, FR, FR, FR, HR, HU, IE, LU, NL, PL, PT, RO, SI, SK, UK
Mesotrione	other	Triketone herbicides	C14H13NO7S	HRAC/WSSA 27	16 cases, 3 countries, 4 species	III	01/06/2017	31/05/2032	AT, BE, BG, CY, CZ, DE, DK, EE, EL, ES, FR, FR, FR, HR, HU, IE, IT, LT, LU, LV, MT, NL, PL, PT, RO, SE, SI, SK, UK
Sulcotrione	other	Triketone herbicides	C14H13ClO5S	HRAC/WSSA 27	*Not available*		01/09/2009	30/11/2026	BE, BG, CZ, DE, EL, ES, FR, FR, FR, FR, HU, LU, NL, PL, PT, RO, SK
Tembotrione	other	Triketone herbicides	C17H16ClF3O6S	HRAC/WSSA 27	10 cases, 2 countries, 3 species		01/05/2014	31/07/2024	AT, BE, BG, CY, CZ, DE, EL, ES, FR, FR, FR, HR, HU, LU, NL, PL, PT, RO, SI, SK, UK
Aclonifen	other	Diphenyl ether herbicides	C12H9ClN2O3	HRAC/WSSA 32	*Not available*	U	01/08/2009	31/10/2026	AT, BE, BG, CY, CZ, DE, DK, EE, EL, ES, FI, FR, FR, FR, HR, HU, IE, IT, LT, LU, LV, NL, PL, PT, RO, SE, SI, SK, UK
